# Supplementary material for: Myrtinols A–F: New Anti-Inflammatory Peltogynoid Flavonoid Derivatives from the Leaves of Australian Indigenous Plant Backhousia myrtifolia
Source: Molecules. 2023 Feb 25;28(5):2160. doi: 10.3390/molecules28052160 (PMC10004088; doi:10.3390/molecules28052160)
Supplement: Supplementary file 1 [file molecules-28-02160-s001.zip › Supporting information_myrtinols_revised.pdf]

Supporting Information

Myrtinols A – F: New anti-inflammatory Peltogynoid flavonoid derivatives from the leaves of Australian Indigenous plant *Backhousia myrtifolia*

Shintu Mathew, [†,‡] Kenneth Zhang, [§] Xian Zhou,[†] Gerald Münch[†,‡] Francis Bodkin, [‡] Feng Li, [§] Ritesh Raju,[*,‡]

[†] NICM Health Research Institute, Western Sydney University, Penrith, NSW, Australia.

[‡] Department of Pharmacology, Western Sydney University, Campbelltown Campus, Sydney, Australia

[§] School of Science, Western Sydney University, Penrith, Sydney, NSW, Australia

Table of Contents

Figure S1. ^{1}H NMR spectrum of compound **1** (CD_3OD, 600 MHz) …..7

Figure S2. COSY spectrum of compound **1** (CD_3OD, 600 MHz) 8

Figure S3. HSQC spectrum of compound **1** (CD_3OD, 600 MHz) 9

Figure S4. HMBC spectrum of compound **1** (CD_3OD, 600 MHz) 10

Figure S5. UV-Vis spectrum for compound **1**..11

Figure S6. HRMS of compound **1** 12

Figure S7. ^{1}H NMR spectrum of compound **2** (CD_3OD, 600 MHz) …..13

Figure S8. COSY spectrum of compound **2** (CD_3OD, 600 MHz) 14

Figure S9. HSQC spectrum of compound **2** (CD_3OD, 600 MHz) 15

Figure S10. HMBC spectrum of compound **2** (CD_3OD, 600 MHz) 16

Figure S11. NOESY spectrum of compound **2** (CD_3OD, 600 MHz) 17

Figure S12. UV-Vis spectrum for compound **2**.. 18

Figure S13. HRMS of compound **2**.. 19

Table S1. Crystallographic data for compound **2**.. 20

Figure S14. ^{1}H NMR spectrum of compound **3** (CD_3OD, 600 MHz) 21

Figure S15. COSY spectrum of compound **3** (CD_3OD, 600 MHz) 22

Figure S16. HSQC spectrum of compound **3** (CD_3OD, 600 MHz) 23

Figure S17. HMBC spectrum of compound **3** (CD_3OD, 600 MHz) 24

Figure S18. NOESY spectrum of compound **3** (CD_3OD, 600 MHz) 25

Figure S19. UV-Vis spectrum for compound **3**.. 26

Figure S20. HRMS of compound **3** 27

Figure S21. ^{1}H NMR spectrum of compound **4** (CD_3OD, 600 MHz) 28

Figure S22. COSY spectrum of compound **4** (CD_3OD, 600 MHz) 29

Figure S23. HSQC spectrum of compound **4** (CD_3OD, 600 MHz) 30

Figure S24. HMBC spectrum of compound **4** (CD_3OD, 600 MHz) 31

Figure S25. NOESY spectrum of compound **4** (CD_3OD, 600 MHz) 32

Figure S26. UV-Vis spectrum for compound **4**........33

Figure S27. HRMS of compound **4** 34

Figure S28. ^{1}H NMR spectrum of compound **5** (CD_3OD, 600 MHz) 35

Figure S29. COSY spectrum of compound **5** (CD_3OD, 600 MHz) 36

Figure S30. HSQC spectrum of compound **5** (CD_3OD, 600 MHz) 37

Figure S31. HMBC spectrum of compound **5** (CD_3OD, 600 MHz) 38

Figure S32. NOESY spectrum of compound **2** (CD_3OD, 600 MHz) 39

Figure S33. UV-Vis spectrum for compound **5**...40

Figure S34. HRMS of compound **5** 41

Figure S35. ^1H NMR spectrum of compound **6** ($CDCl_3$, 600 MHz) 42

Figure S36. COSY spectrum of compound **6** ($CDCl_3$, 600 MHz) 43

Figure S37. HSQC spectrum of compound **6** ($CDCl_3$, 600 MHz) 44

Figure S38. HMBC spectrum of compound **6** ($CDCl_3$, 600 MHz) 45

Figure S39. UV-Vis spectrum for compound **6**...46

Figure S40. HRMS of compound **6** 47

Figure S41. ^1H NMR spectrum of compound **7** (DMSO-d_6, 400 MHz) 48

Figure S42. HRMS of compound **7** 49

Figure S43. ^{1}H NMR spectrum of compound **8** (CD_3OD, 600 MHz) 50

Figure S44. HRMS of compound **8** 51

Figure S45. ^{1}H NMR spectrum of compound **9** (DMSO-d_6, 600 MHz) 52

Figure S46. HRMS of compound **9** 53

Table S2. NMR data (600 MHz, CD_3OD-*d_4* for compound **1** 54

Table S3. NMR data (600 MHz, CD_3OD-*d_4* for compound **2** 55

Table S4. NMR data (600 MHz, CD_3OD-*d_4* for compound **3** 56

Table S5. NMR data (600 MHz, CD_3OD-*d_4* for compound **4** 57

Table S6. NMR data (600 MHz, CD_3OD-*d_4* for compound **5** 58

Table S7. NMR data (600 MHz, $CDCl_3$) for compound **6** 59

Table S8. Downregulation of LPS and IFN- γ induced nitric oxide production and cell viability of sequential extract....................................60

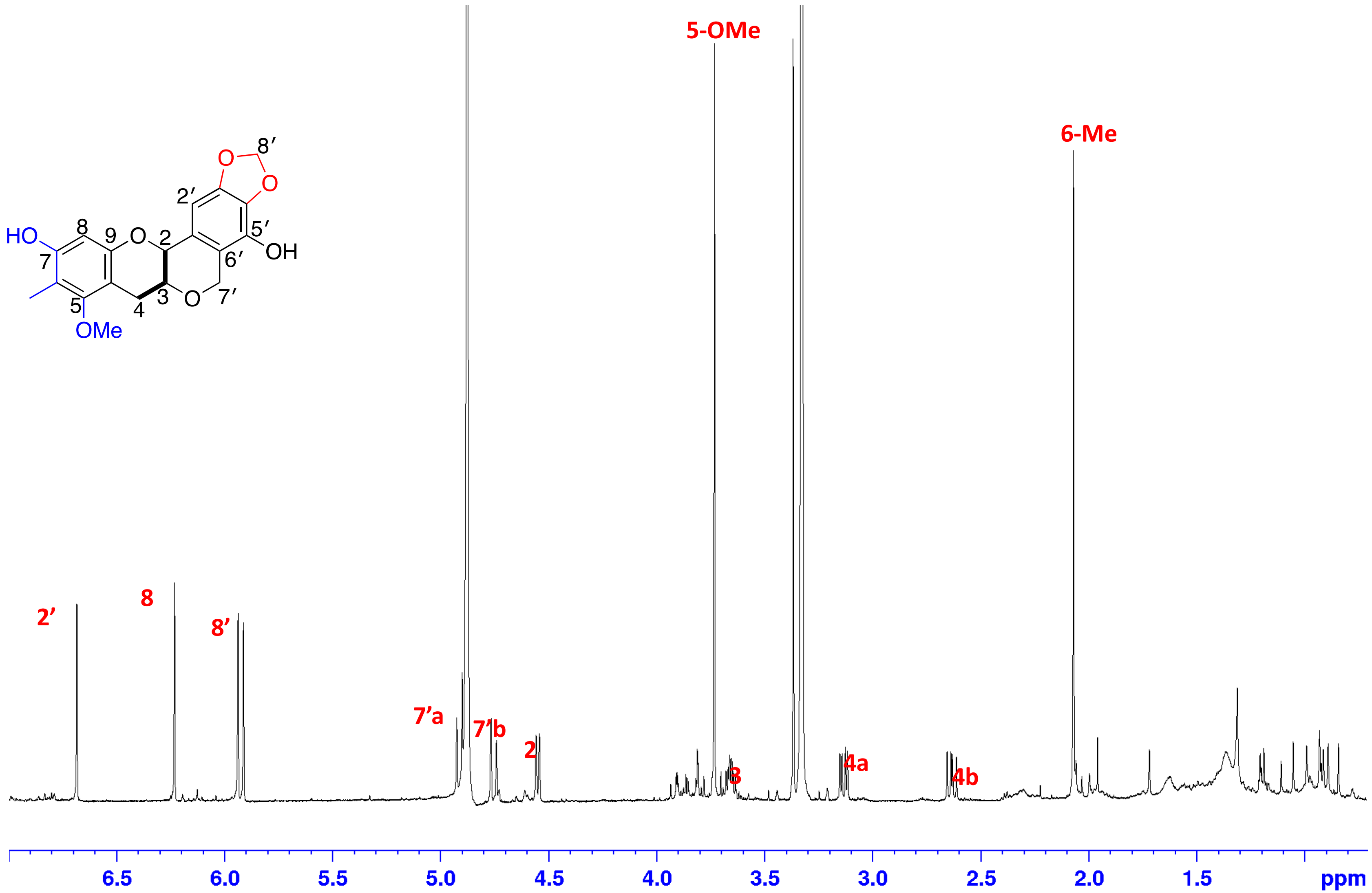

Figure S1. ^{1}H NMR spectrum of compound **1** (CD_3OD, 600 MHz)

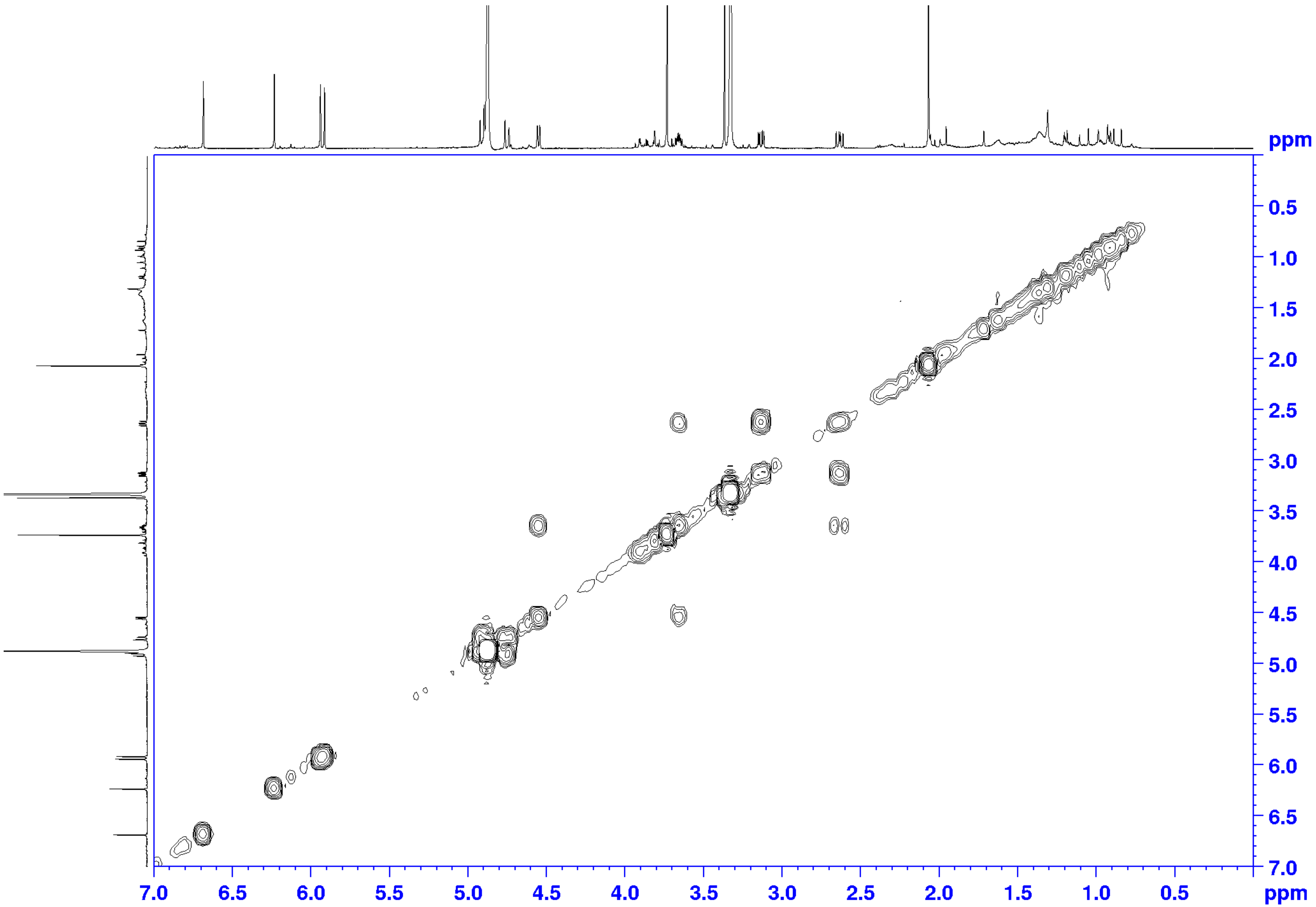

Figure S2. COSY spectrum of compound **1** (CD_3OD, 600 MHz)

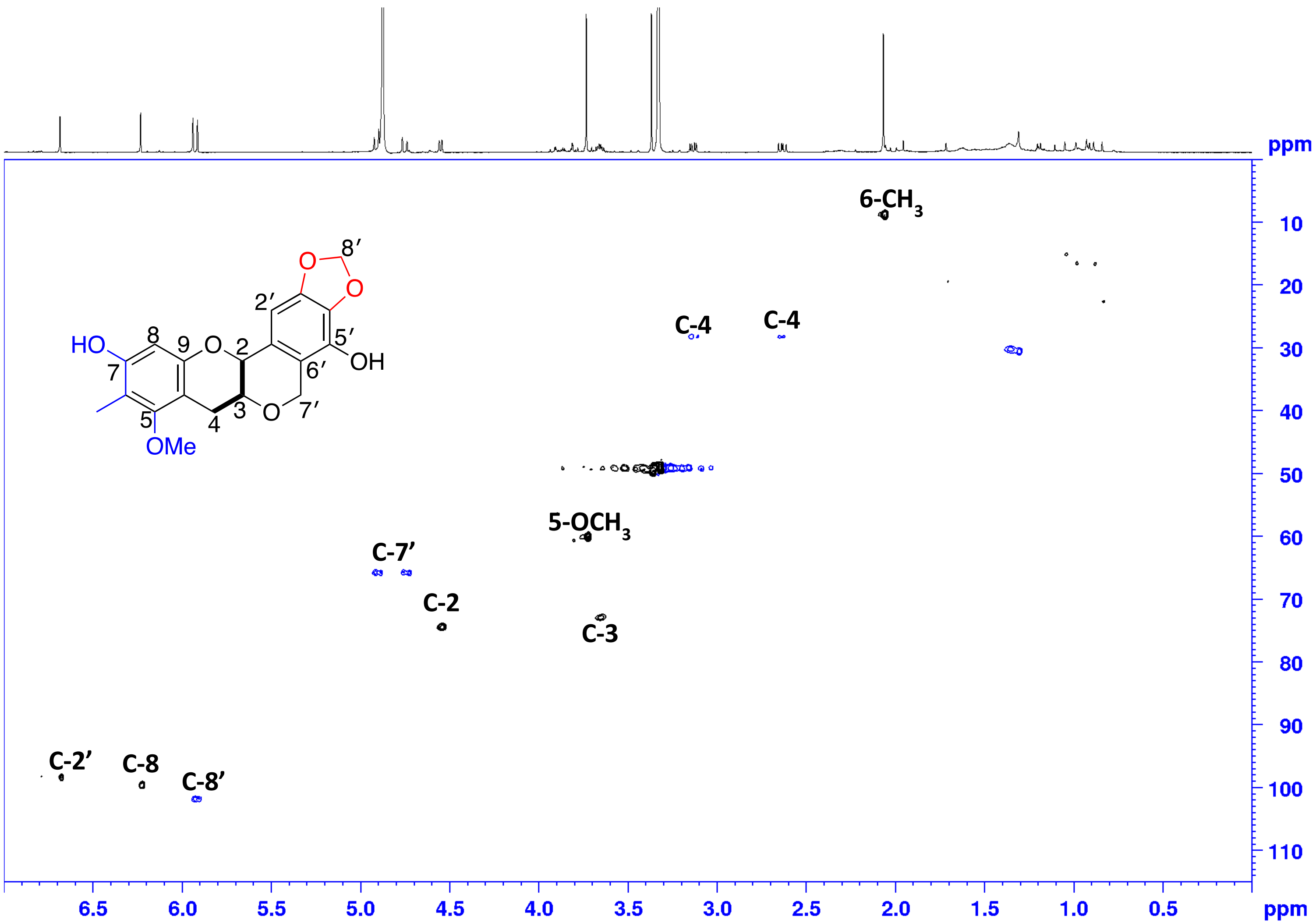

Figure S3. HSQC spectrum of compound **1** (CD_3OD, 600 MHz)

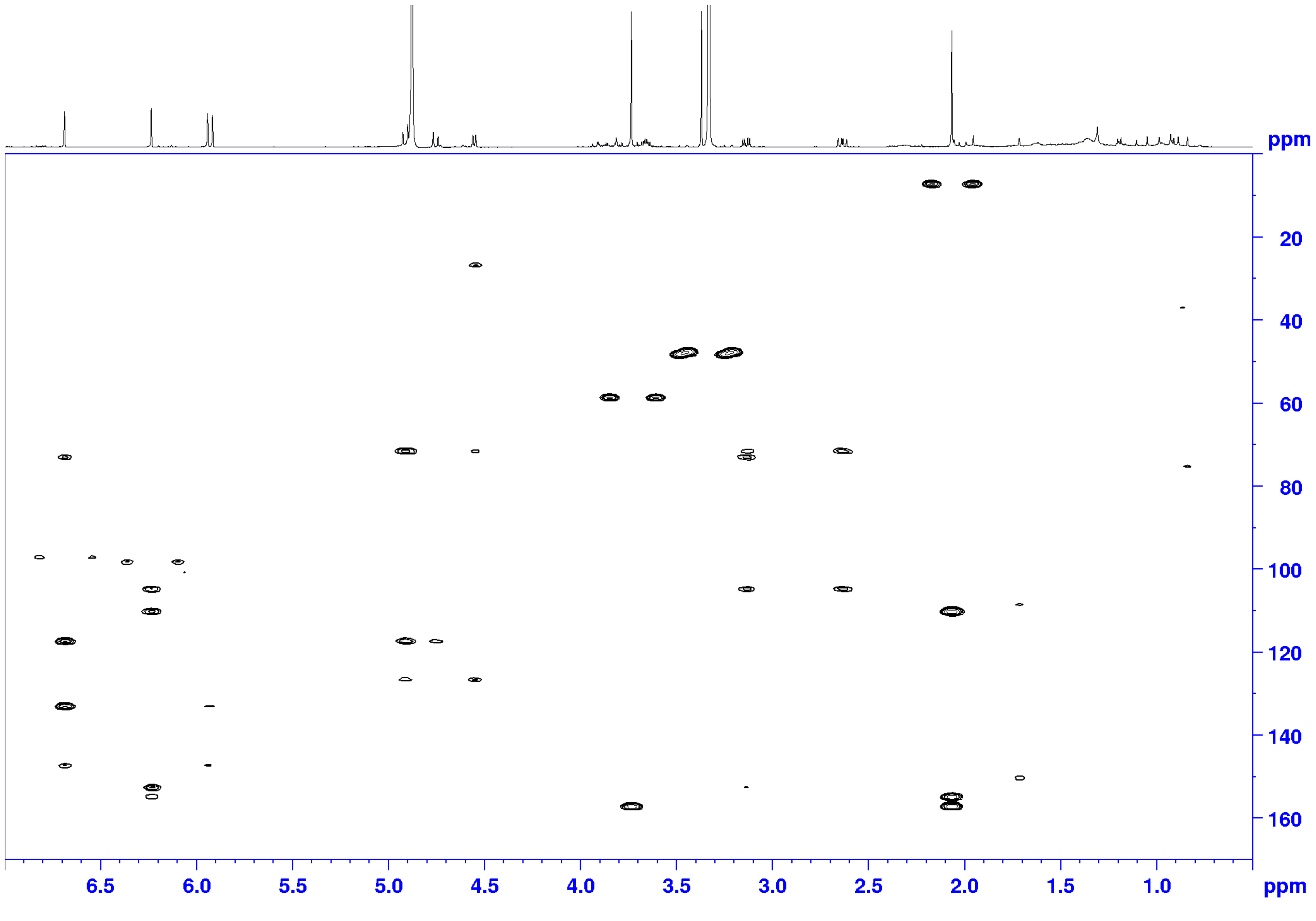

Figure S4. HMBC spectrum of compound **1** (CD_3OD, 600 MHz)

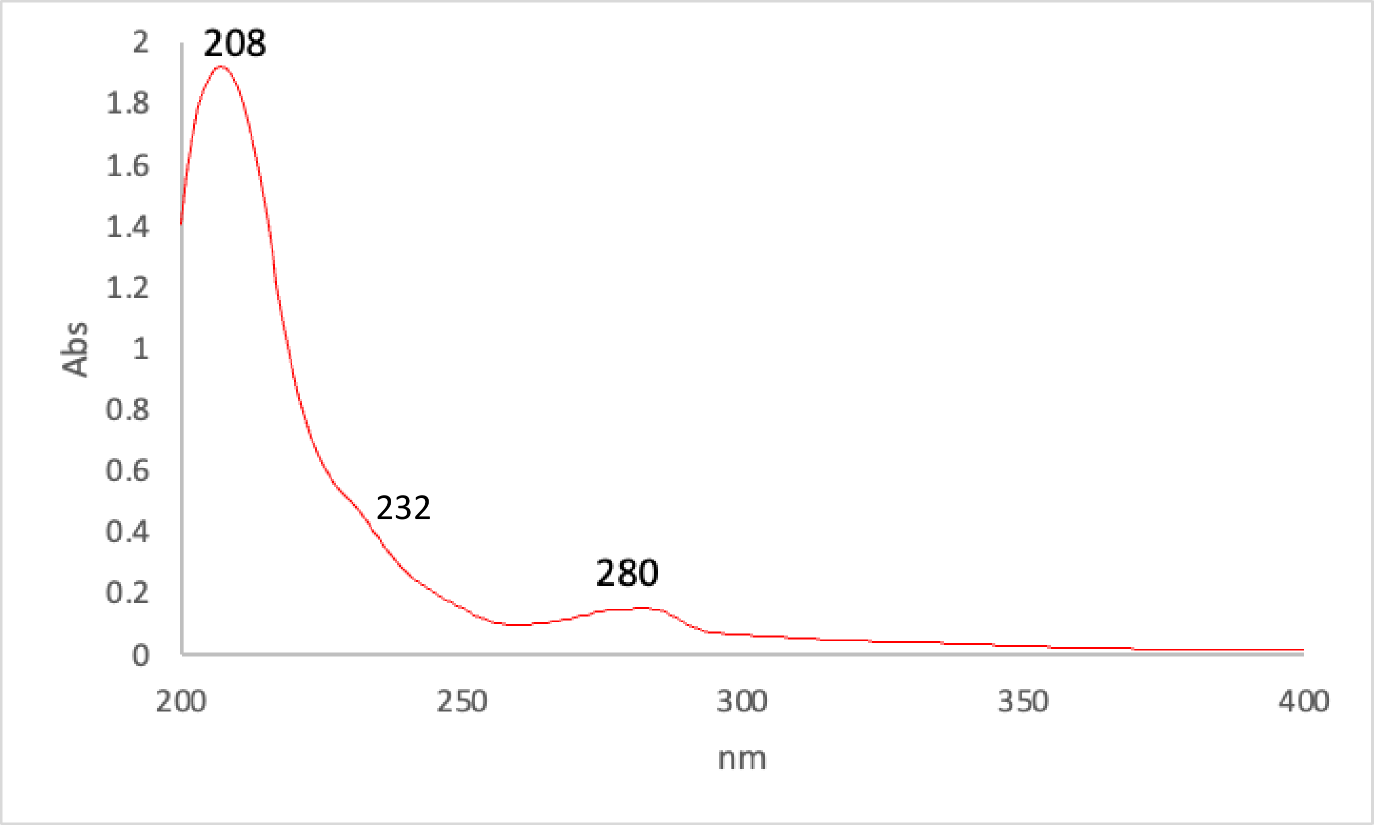

Figure S5. UV-Vis spectrum for compound **1**

Elemental Composition Report

Single Mass Analysis

Tolerance = 5.0 PPM / DBE: min = -1.5, max = 50.0
Element prediction: Off
Number of isotope peaks used for i-FIT = 9

Monoisotopic Mass, Even Electron Ions
17 formula(e) evaluated with 1 results within limits (all results (up to 1000) for each mass)
Elements Used:
C: 0-25 H: 0-30 O: 0-8

011222_BMF-8 124 (2.430) Cm (124)
1: TOF MS ES-

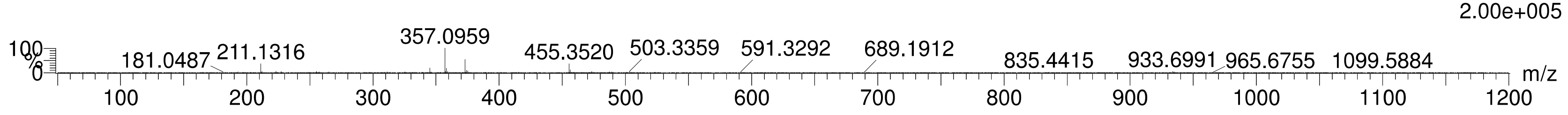

Minimum:				-1.5				
Maximum:		20.0	5.0	50.0				
Mass	Calc. Mass	mDa	PPM	DBE	i-FIT	Norm	Conf(%)	Formula
357.0959	357.0974	-1.5	-4.2	11.5	961.0	n/a	n/a	C19 H17 O7

Figure S6. HRMS of compound **1**

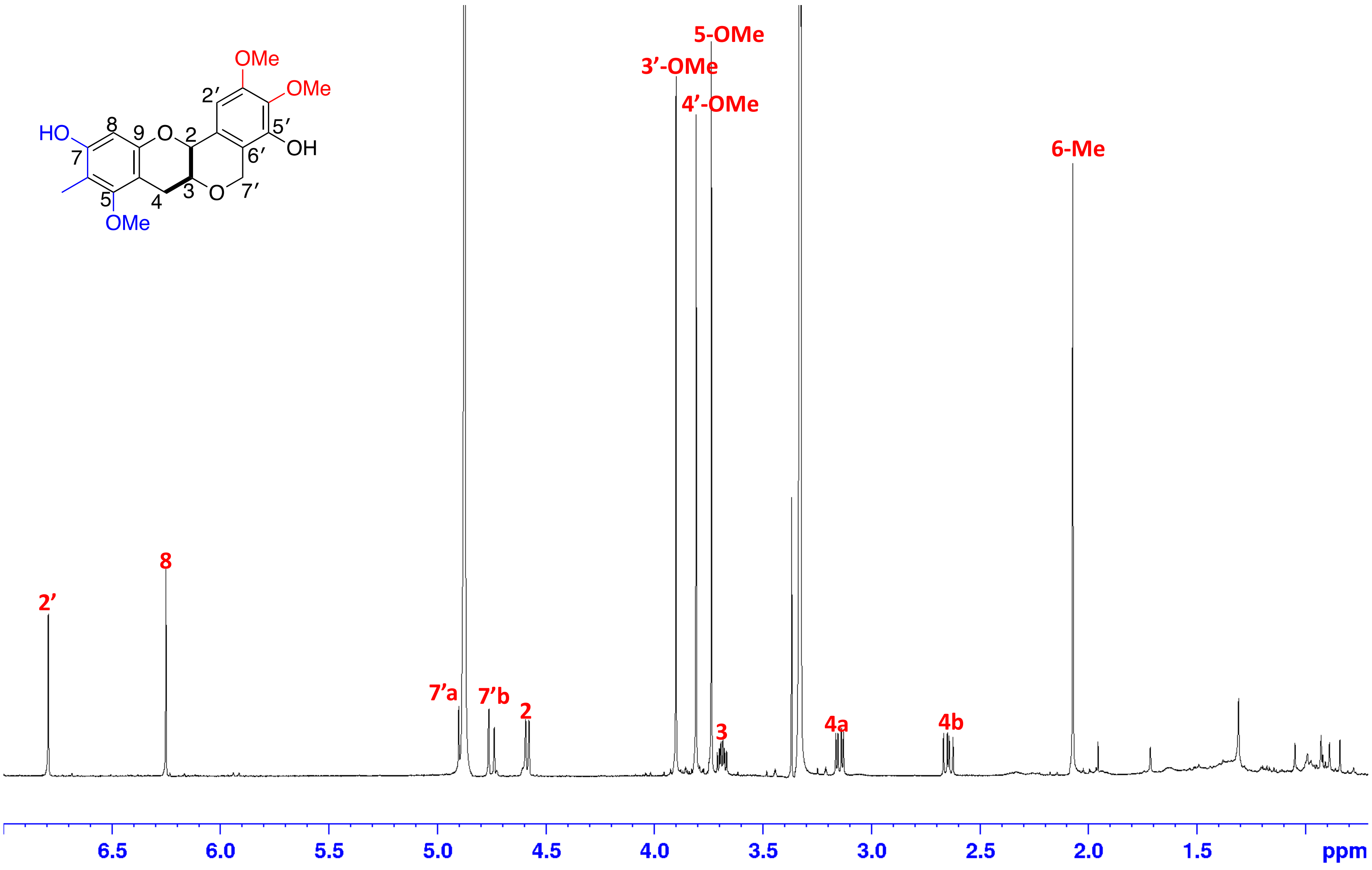

Figure S7. ^{1}H NMR spectrum of compound **2** (CD_3OD, 600 MHz)

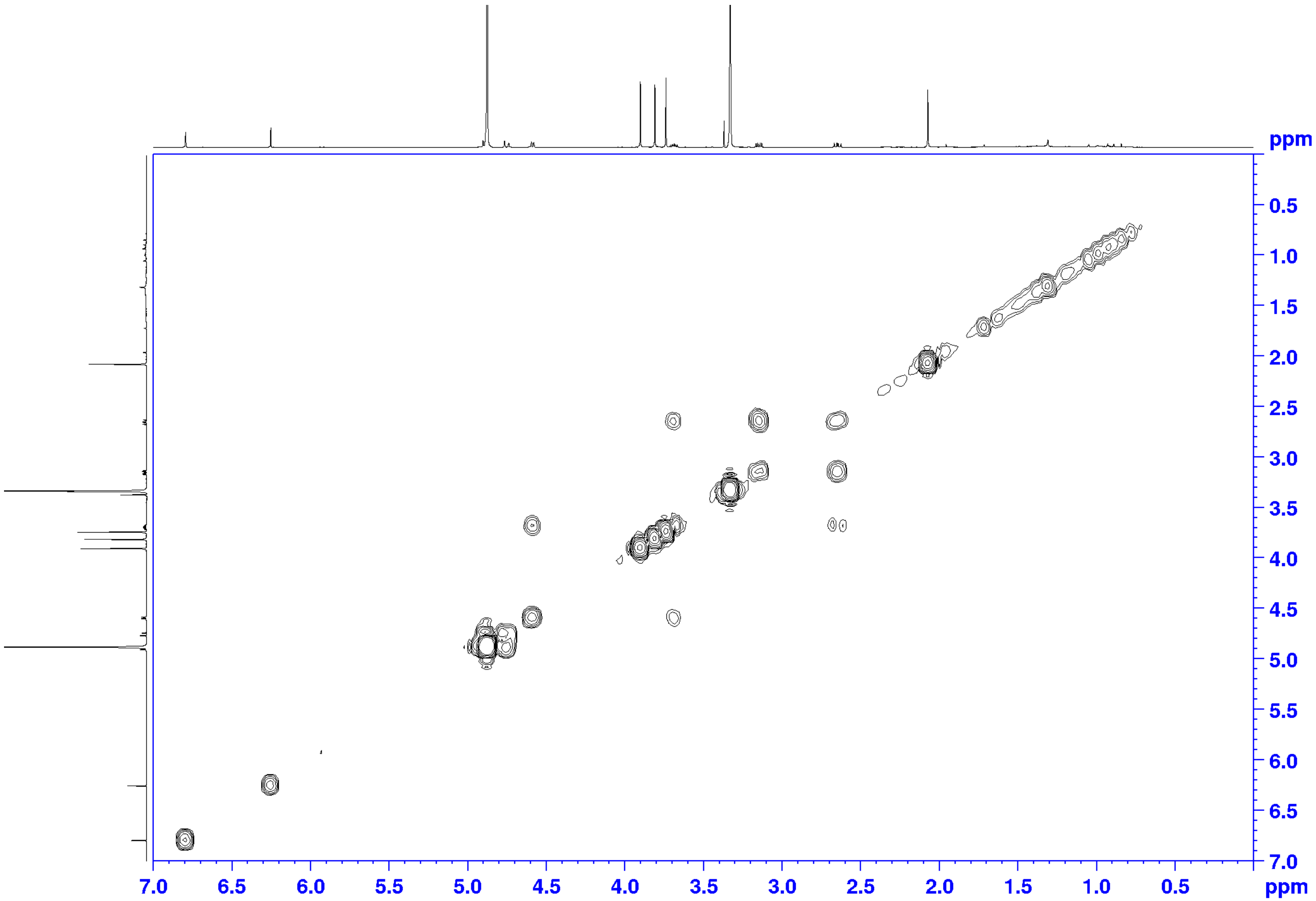

Figure S8. COSY spectrum of compound **2** (CD_3OD, 600 MHz)

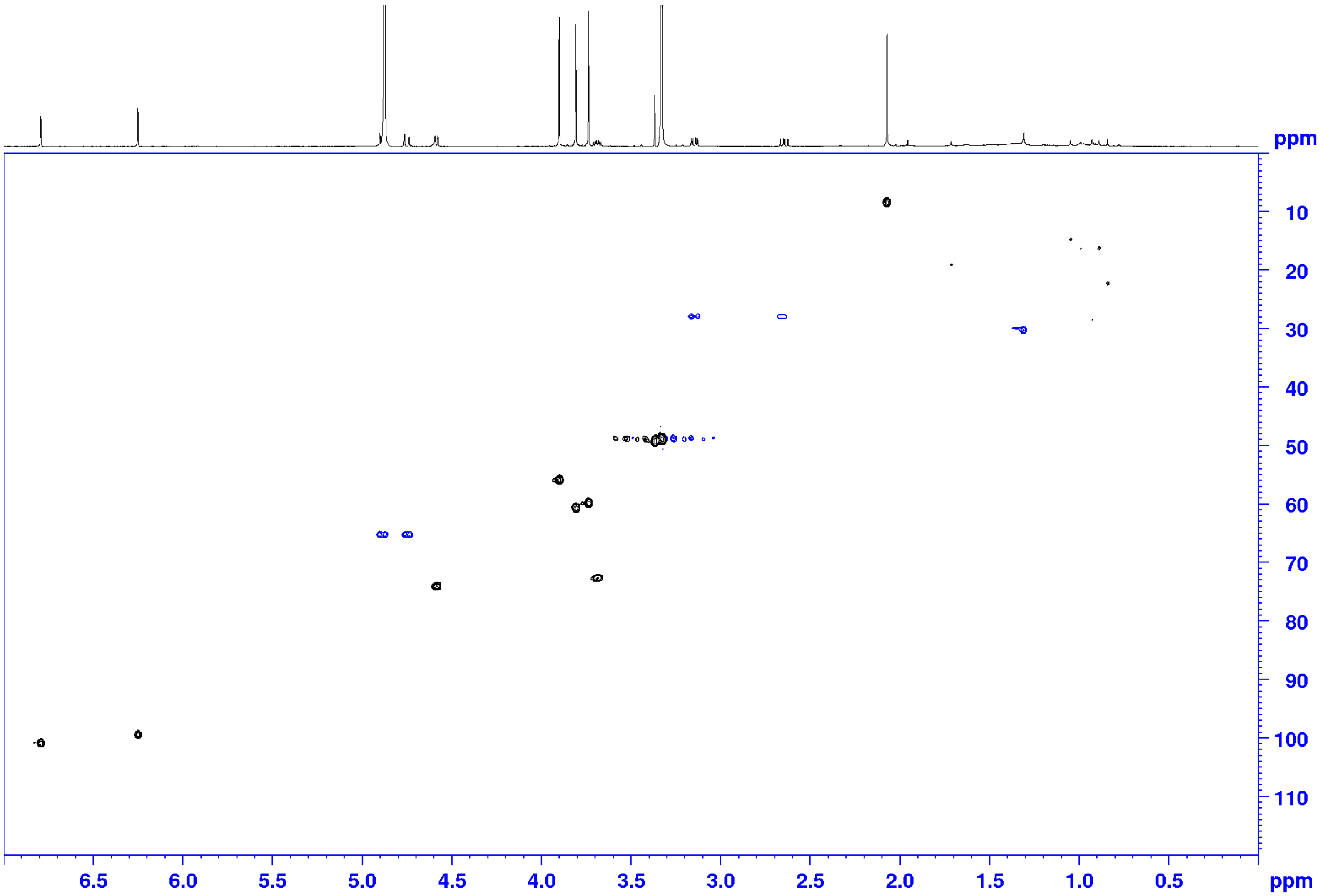

Figure S9. HSQC spectrum of compound **2** (CD_3OD, 600 MHz)

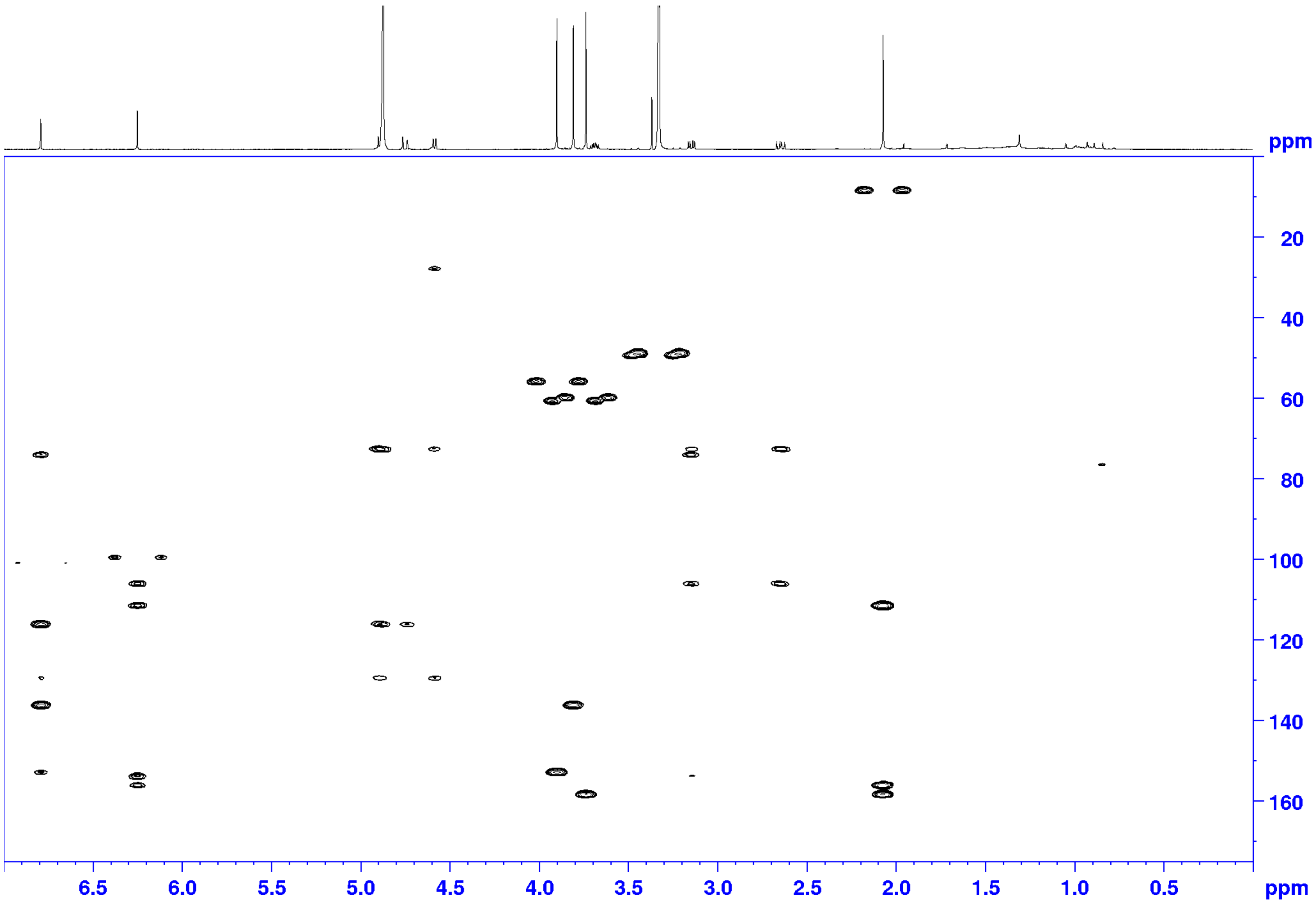

Figure S10. HMBC spectrum of compound **2** (CD_3OD, 600 MHz)

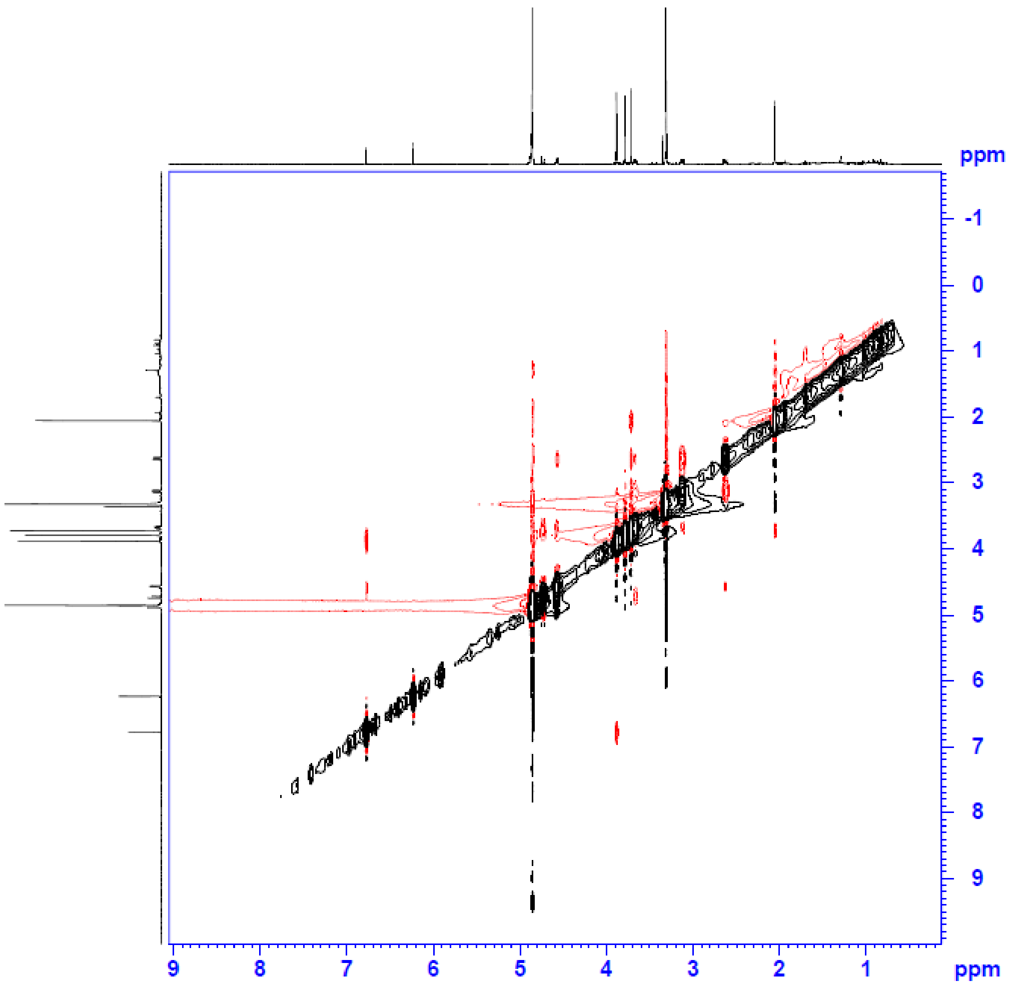

Figure S11. NOESY spectrum of compound **2** (CD_3OD, 600 MHz)

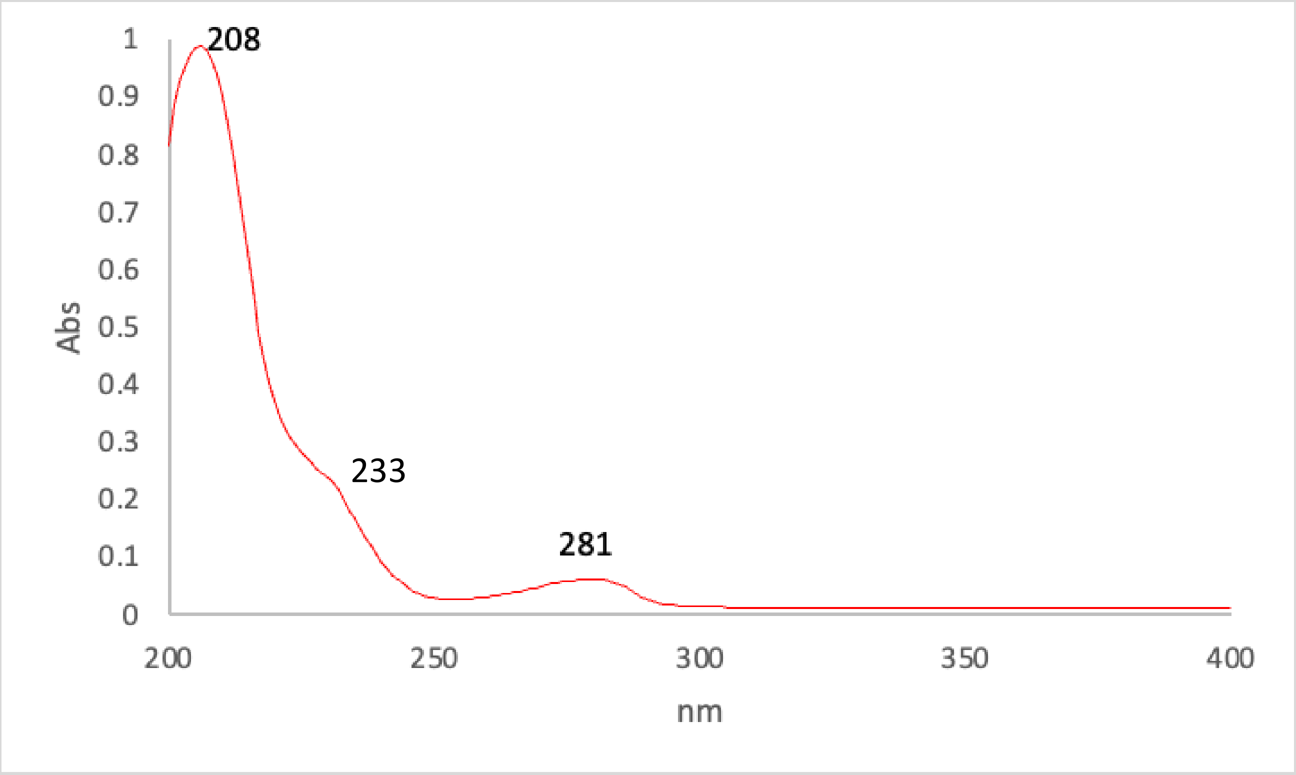

Figure S12. UV-Vis spectrum for compound **2**

Elemental Composition Report

Single Mass Analysis

Tolerance = 10.0 PPM / DBE: min = -1.5, max = 50.0
Element prediction: Off
Number of isotope peaks used for i-FIT = 9

Monoisotopic Mass, Even Electron Ions
16 formula(e) evaluated with 1 results within limits (all results (up to 1000) for each mass)
Elements Used:
C: 0-20 H: 0-26 O: 0-8 Na: 0-1
220829_BM_F9 68 (1.345) Cm (67:80)
1: TOF MS ES+

3.15e+007

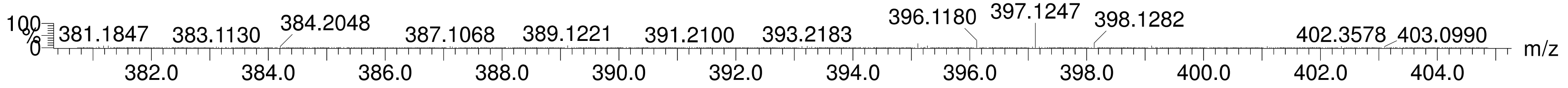

Minimum:				-1.5				
Maximum:		20.0	10.0	50.0				
Mass	Calc. Mass	mDa	PPM	DBE	i-FIT	Norm	Conf(%)	Formula
397.1247	397.1263	-1.6	-4.0	9.5	6422.9	n/a	n/a	C20 H22 O7 Na

Figure S13. HRMS of compound **2**

Table S1. Crystallographic data for compound **2**

CCDC number	2236594
Empirical formula	$C_{20}H_{21}O_7$
Formula weight	373.37
Temperature/K	100.0
Crystal system	triclinic
Space group	P1
a/Å	8.7800(18)
b/Å	8.9200(18)
c/Å	11.620(2)
α/°	84.56(3)
β/°	81.25(3)
γ/°	76.39(3)
Volume/Å^3	872.6(3)
Z	2
ρ_{calc}g/cm^3	1.421
μ/mm^{-1}	0.108
F(000)	394.0
Crystal size/mm^3	0.2 × 0.1 × 0.1
Radiation	MoKα (λ = 0.71073)
2Θ range for data collection/°	3.552 to 57.202
Index ranges	$-11 \leq h \leq 11$, $-11 \leq k \leq 11$, $-15 \leq l \leq 15$
Reflections collected	20884
Independent reflections	6838 [R_{int} = 0.0191, R_{sigma} = 0.0186]
Data/restraints/parameters	6838/3/499
Goodness-of-fit on F^2	1.048
Final R indexes [I>=2σ (I)]	R_1 = 0.0383, wR_2 = 0.1031
Final R indexes [all data]	R_1 = 0.0387, wR_2 = 0.1045
Largest diff. peak/hole / e Å^{-3}	0.36/-0.56
Flack parameter	0.06(13)

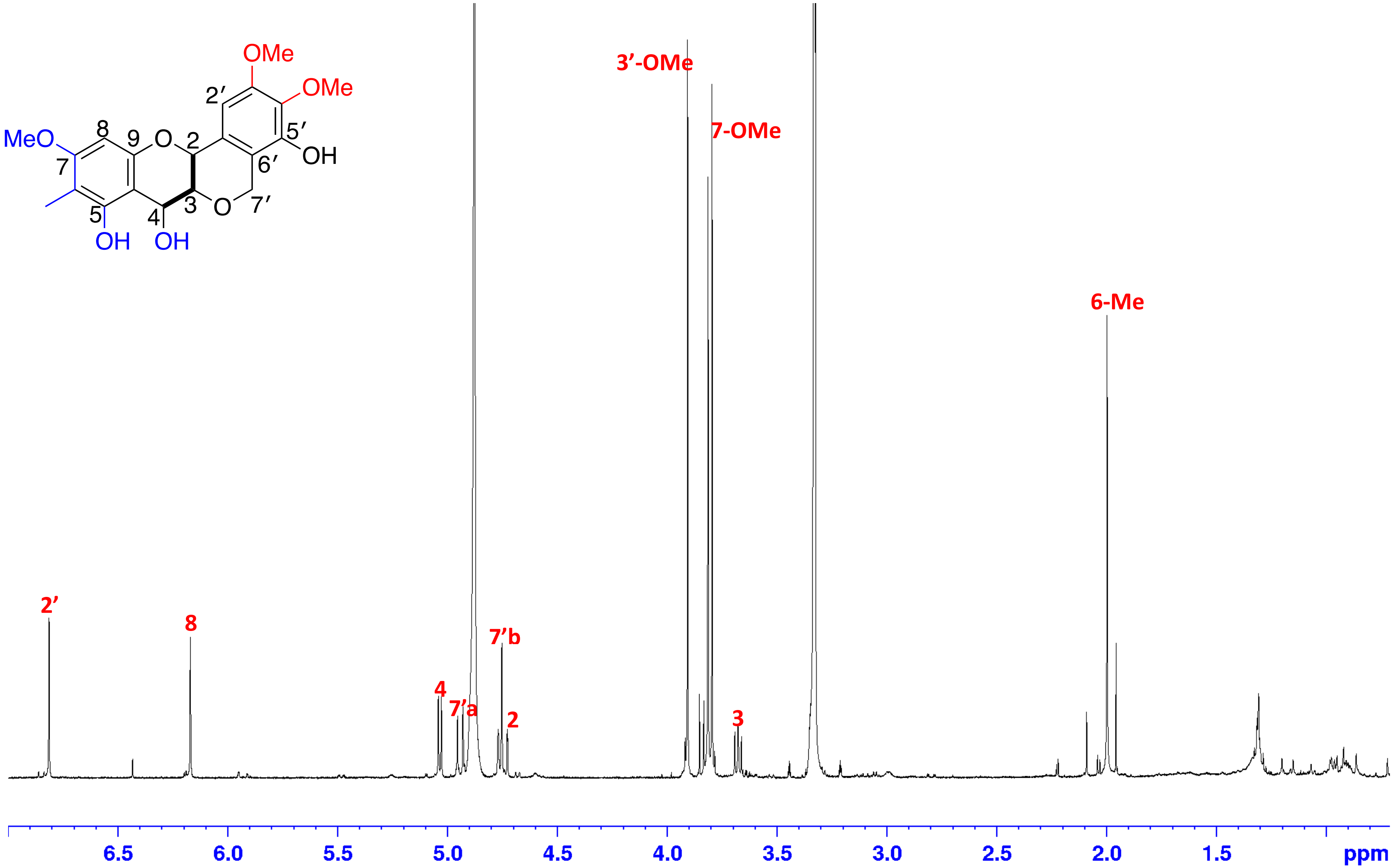

Figure S14. ^{1}H NMR spectrum of compound **3** (CD_3OD, 600 MHz)

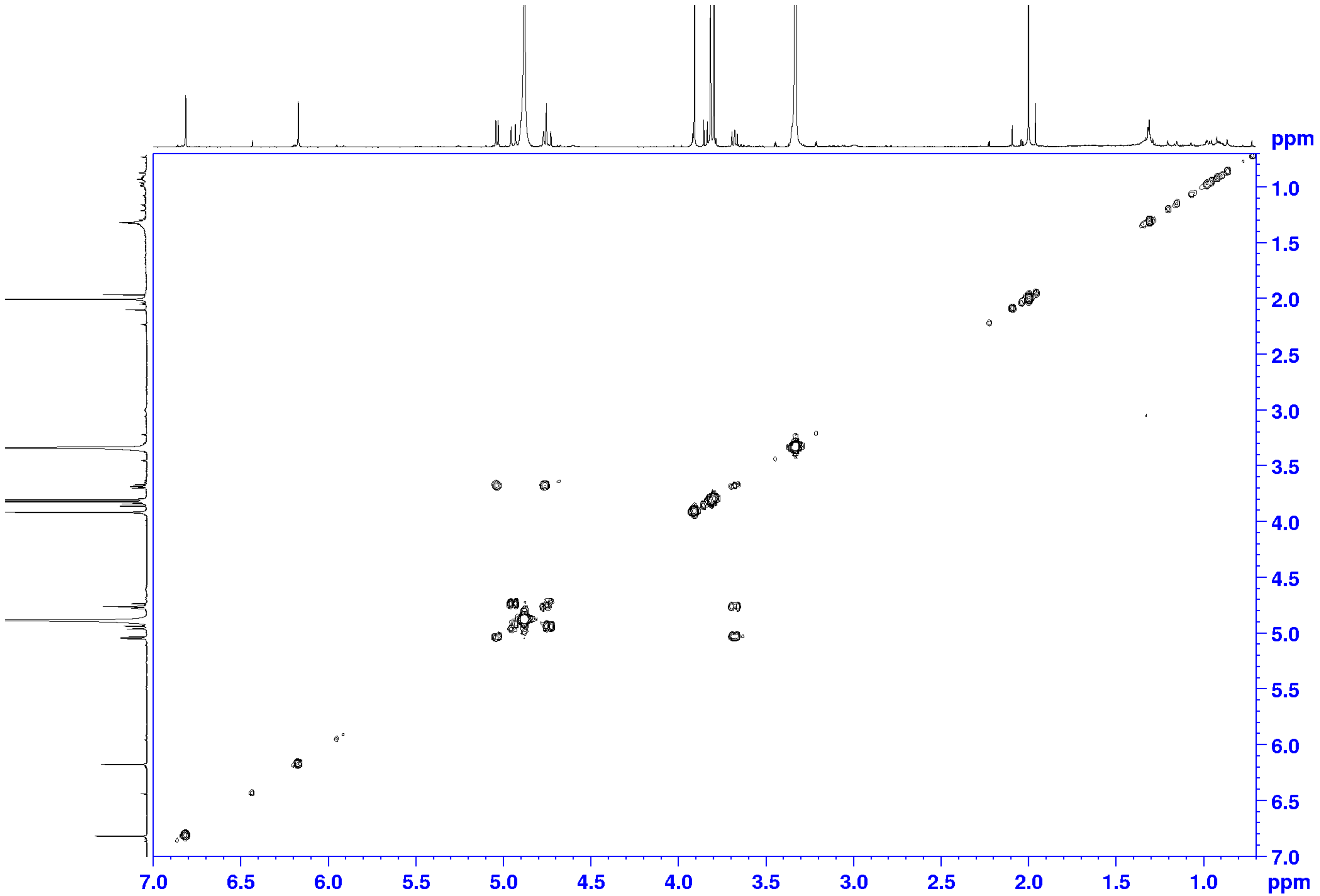

Figure S15. COSY spectrum of compound **3** (CD_3OD, 600 MHz)

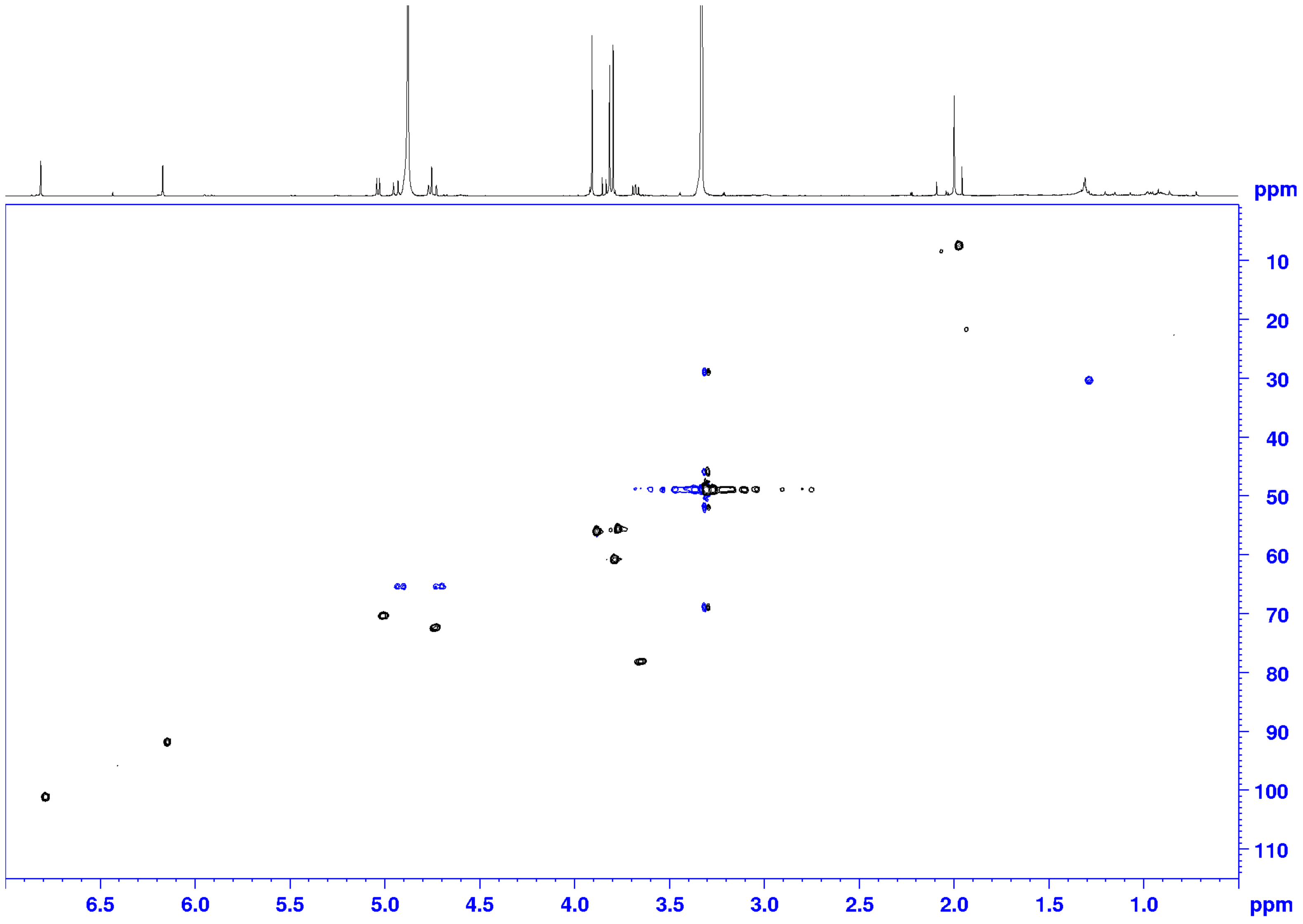

Figure S16. HSQC spectrum of compound **3** (CD_3OD, 600 MHz)

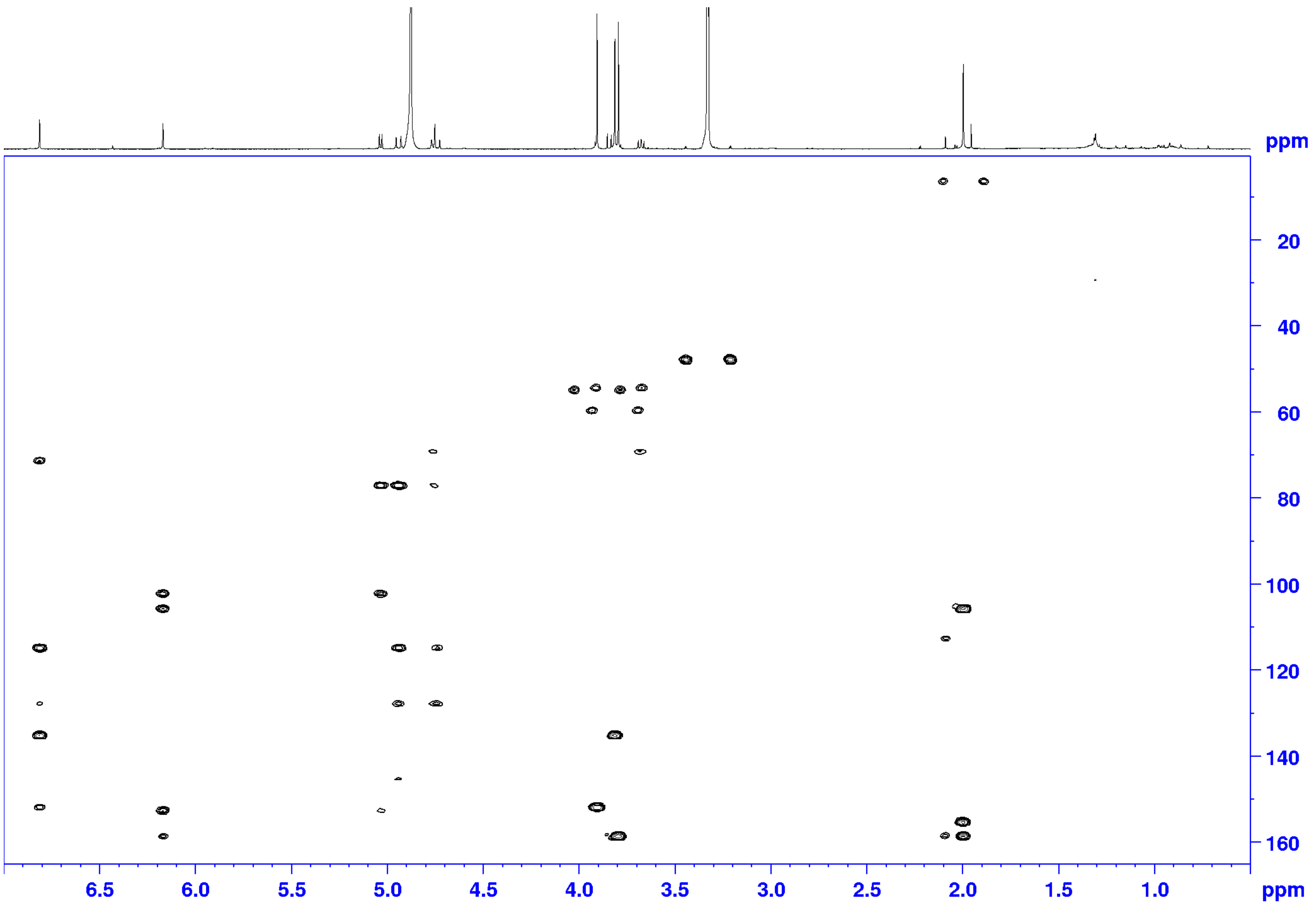

Figure S17. HMBC spectrum of compound **3** (CD_3OD, 600 MHz)

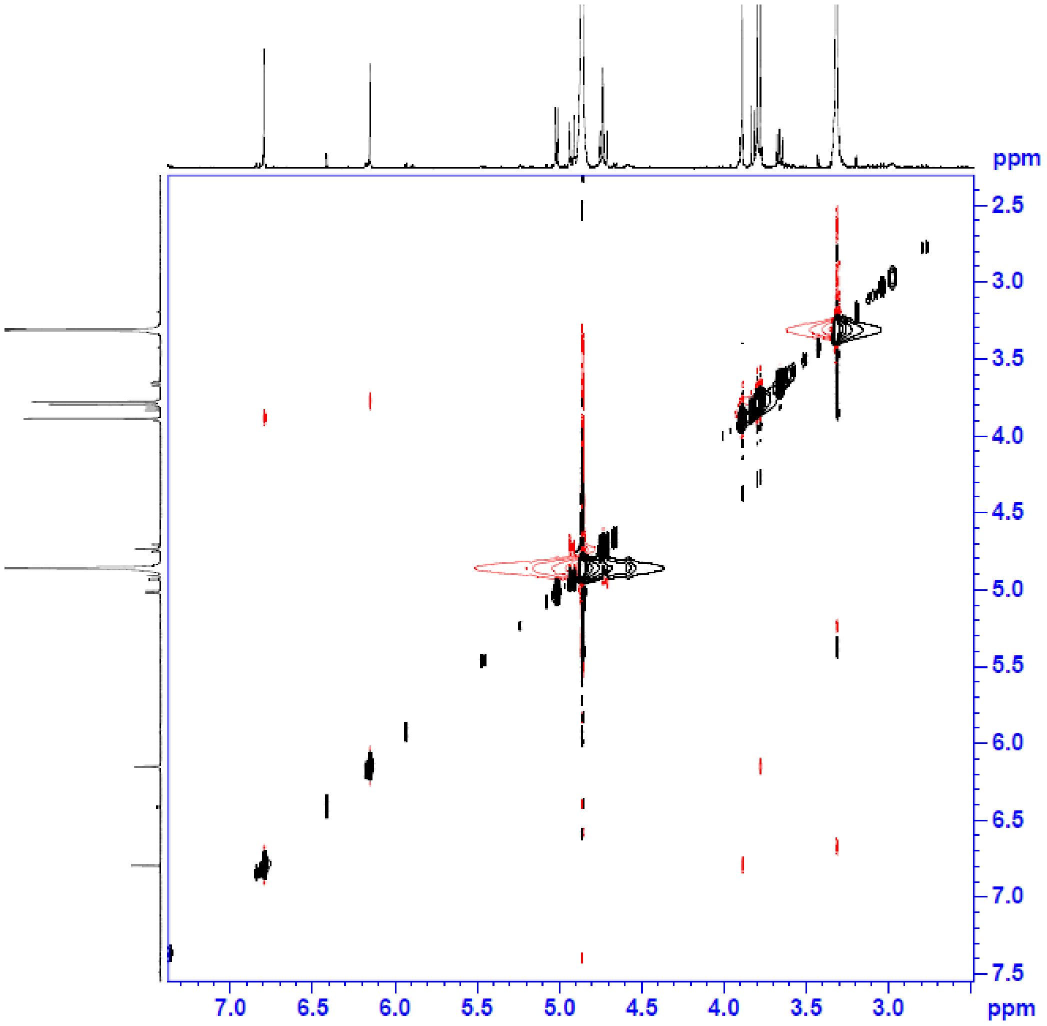

Figure S18. NOESY spectrum of compound **3** (CD_3OD, 600 MHz)

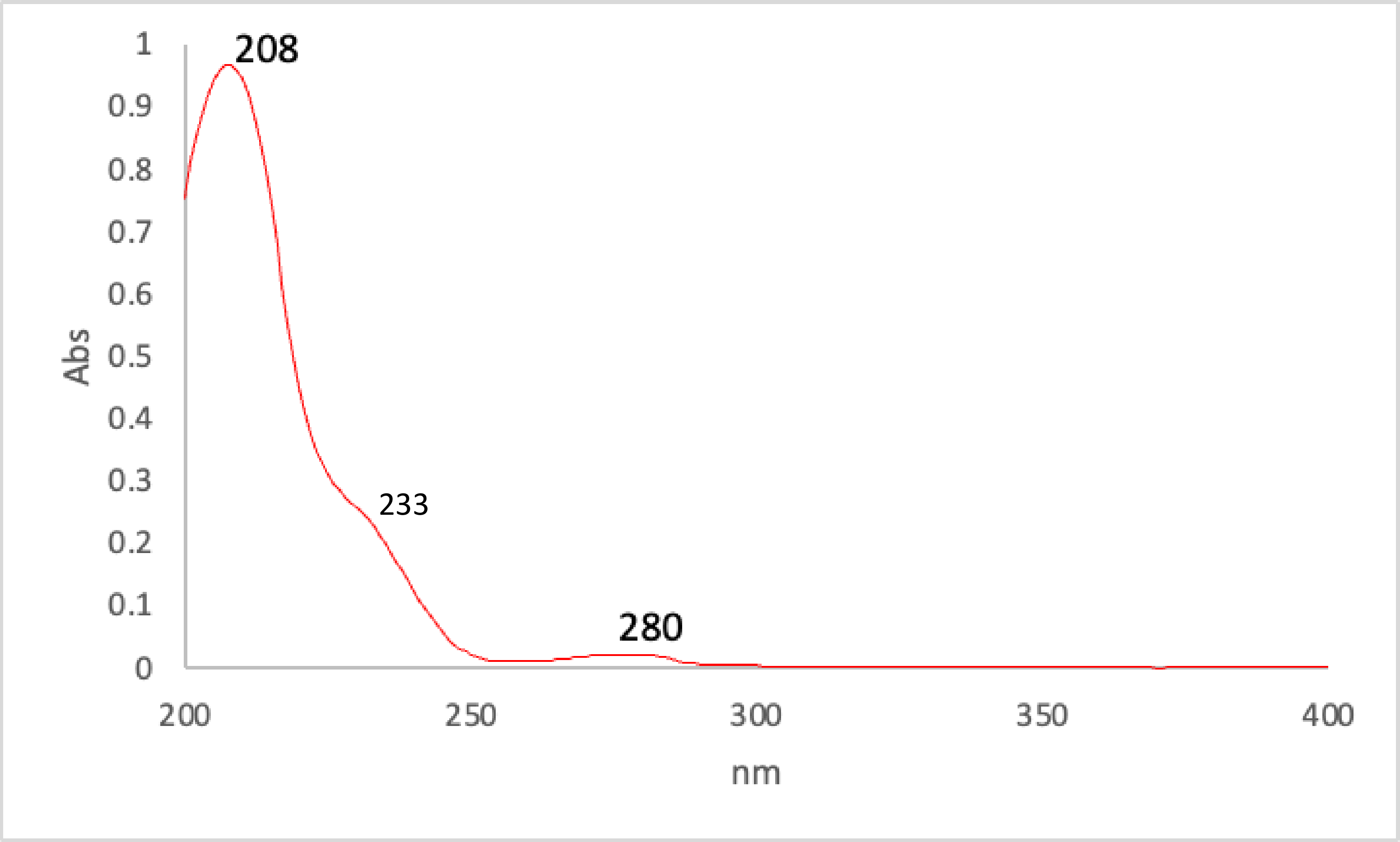

Figure S19. UV-Vis spectrum for compound **3**

Single Mass Analysis

Tolerance = 5.0 PPM / DBE: min = -1.5, max = 50.0
Element prediction: Off
Number of isotope peaks used for i-FIT = 9

Monoisotopic Mass, Even Electron Ions
13 formula(e) evaluated with 1 results within limits (all results (up to 1000) for each mass)
Elements Used:
C: 0-25 H: 0-30 O: 0-8
011222_BMF-11A 123 (2.413) Cm (123)
1: TOF MS ES-

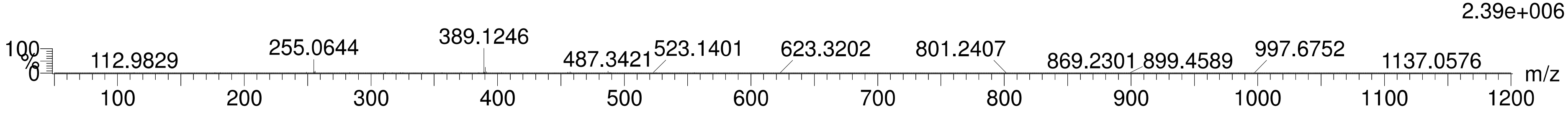

	Mass	Calc. Mass	mDa	PPM	DBE	i-FIT	Norm	Conf(%)	Formula
Minimum:					-1.5				
Maximum:			20.0	5.0	50.0				
	389.1246	389.1236	1.0	2.6	10.5	2068.4	n/a	n/a	C20 H21 O8

Figure S20. HRMS spectrum for compound **3**

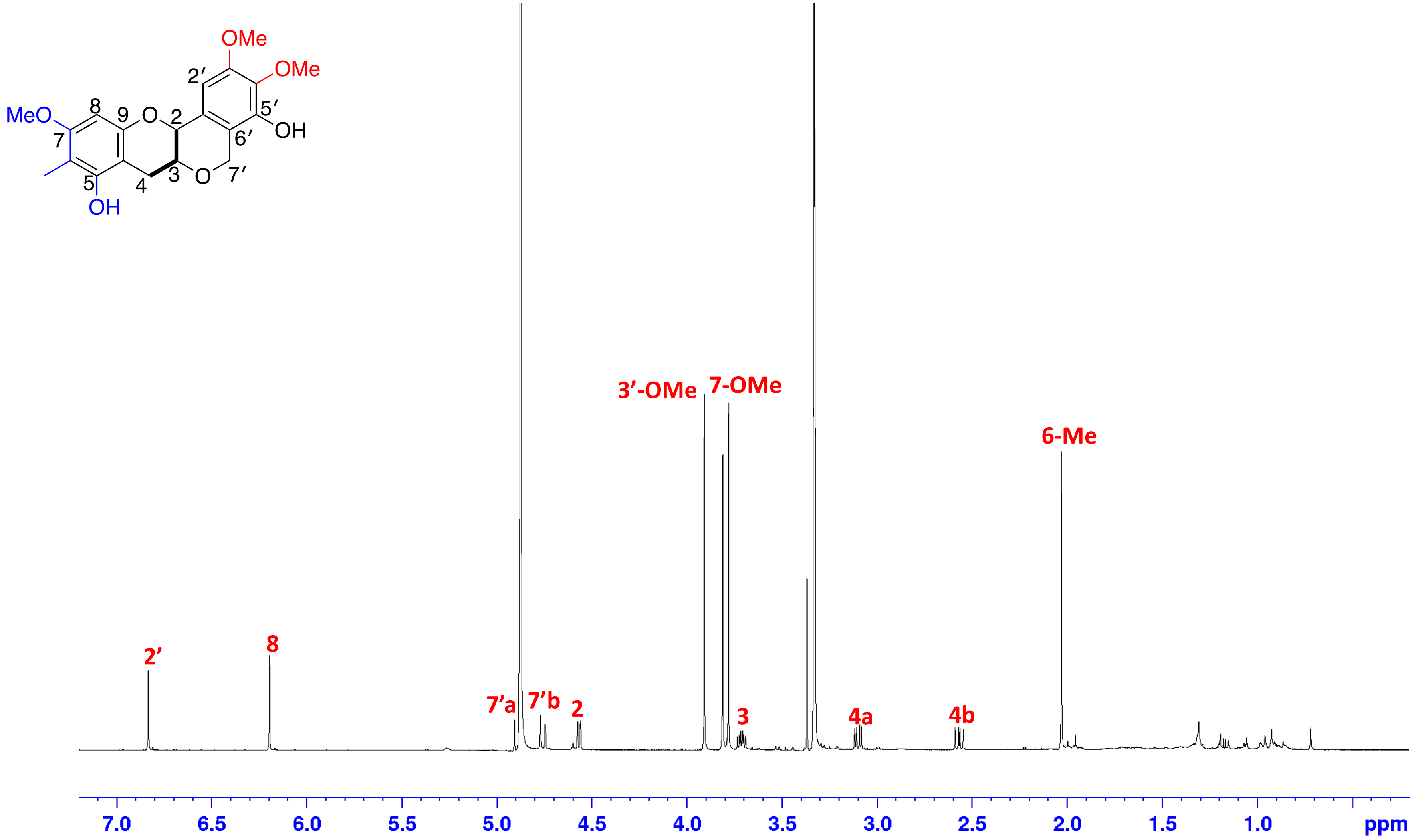

Figure S21. ^{1}H NMR spectrum of compound **4** (CD_3OD, 600 MHz)

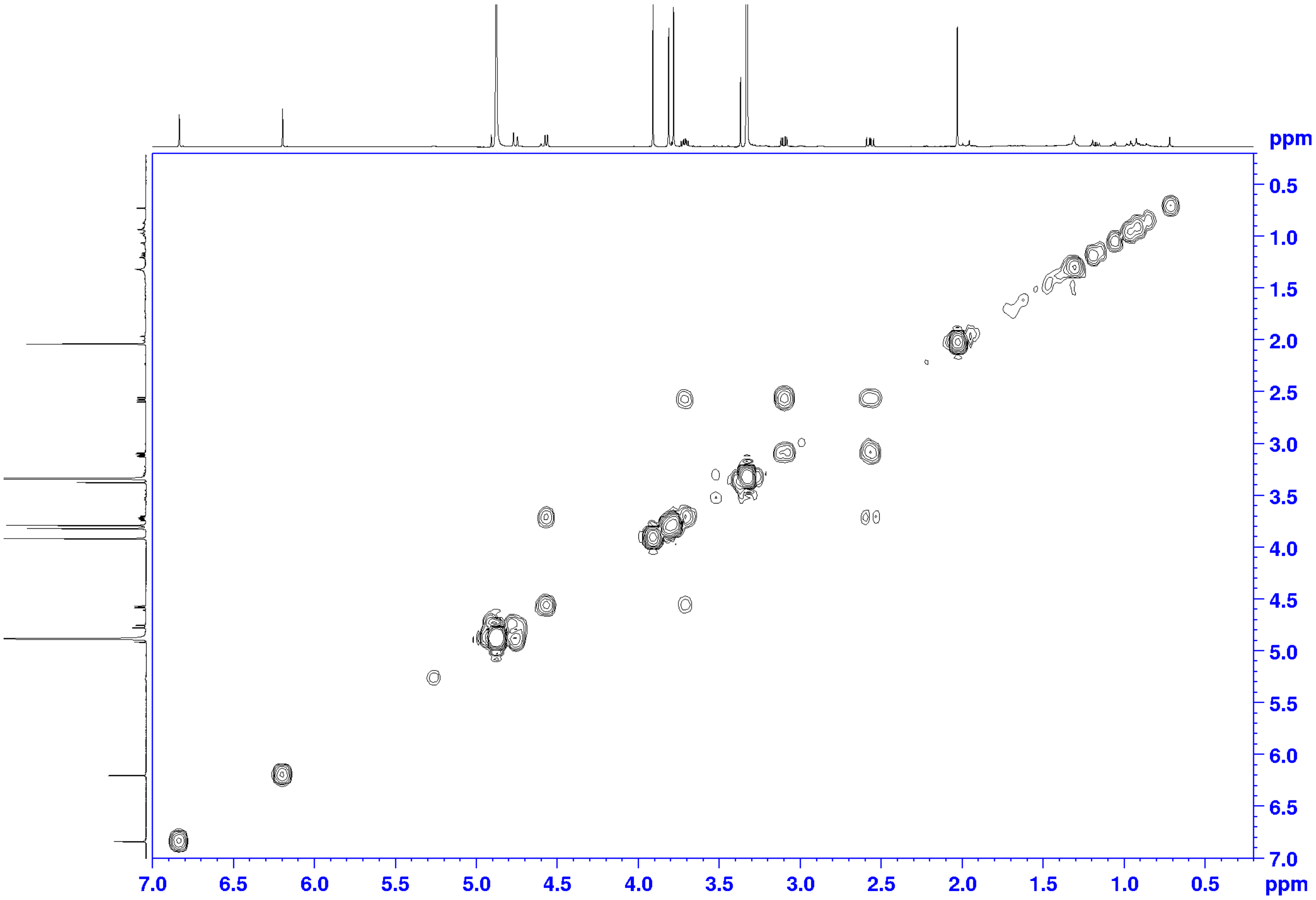

Figure S22. COSY spectrum of compound **4** (CD_3OD, 600 MHz)

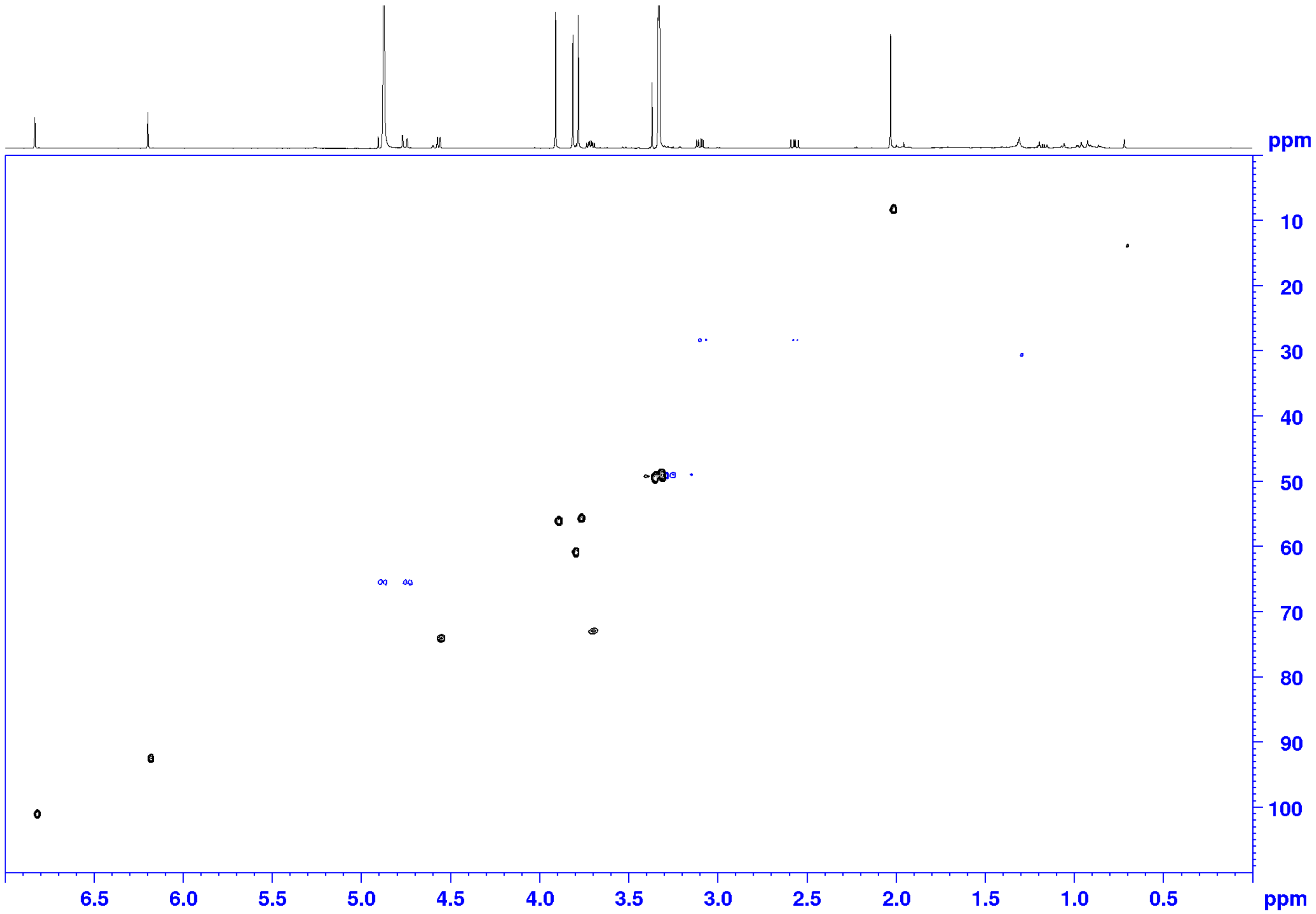

Figure S23. HSQC spectrum of compound **4** (CD_3OD, 600 MHz)

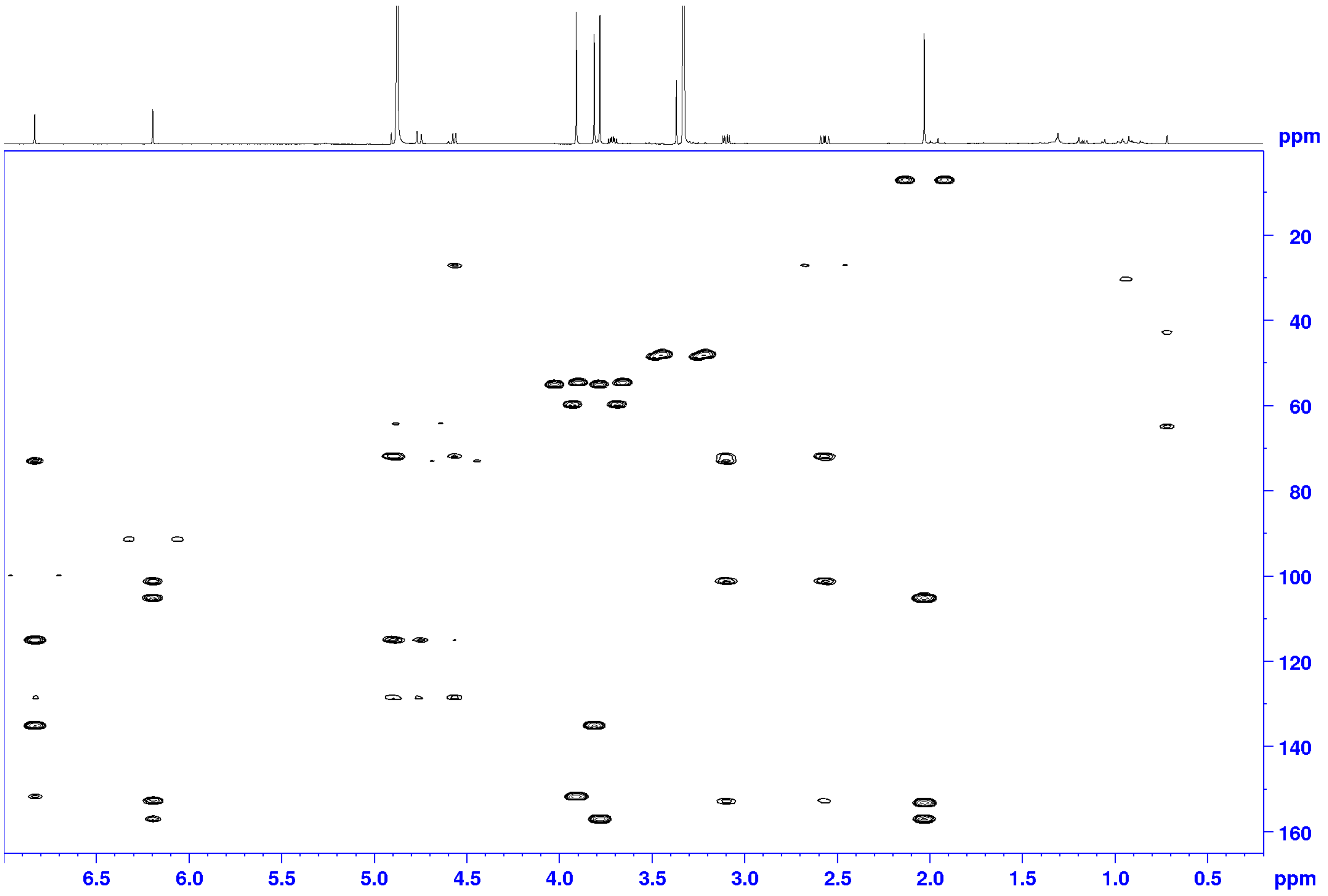

Figure S24. HMBC spectrum of compound **4** (CD_3OD, 600 MHz)

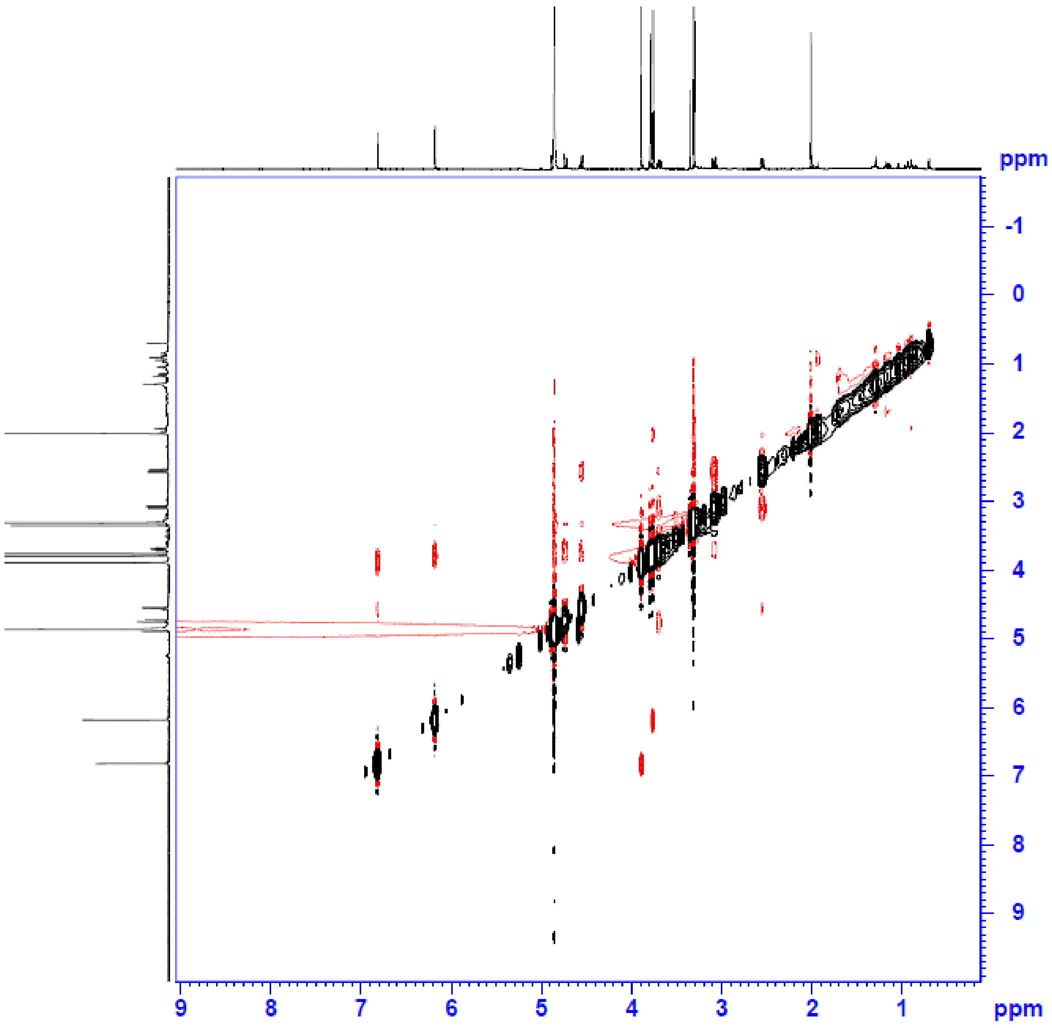

Figure S25. NOESY spectrum of compound **4** (CD_3OD, 600 MHz)

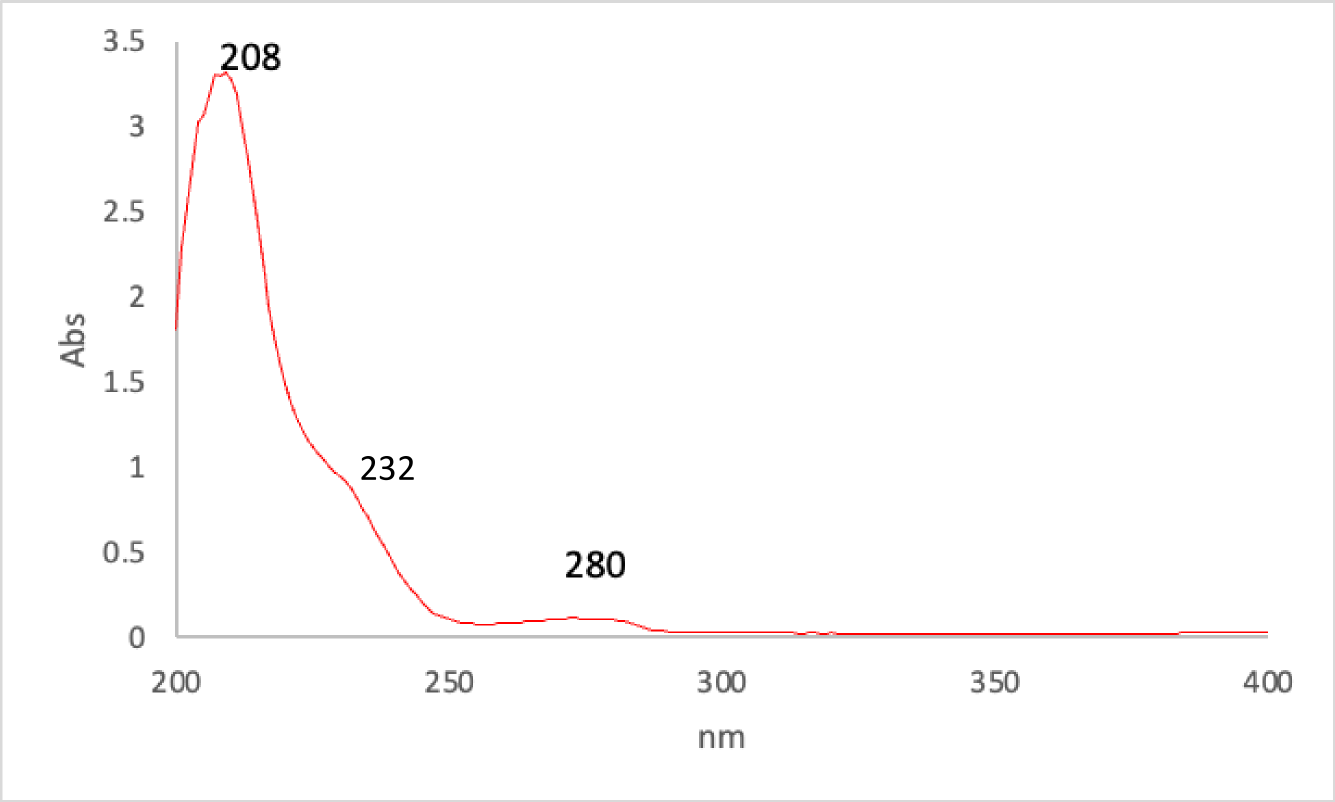

Figure S26. UV-Vis spectrum for compound **4**

Elemental Composition Report

Single Mass Analysis

Tolerance = 10.0 PPM / DBE: min = -1.5, max = 50.0
Element prediction: Off
Number of isotope peaks used for i-FIT = 9

Monoisotopic Mass, Even Electron Ions
9 formula(e) evaluated with 1 results within limits (all results (up to 1000) for each mass)
Elements Used:
C: 0-20 H: 0-22 O: 0-8
221103_BM_F11B_neg 17 (0.364) Cm (15:17)
1: TOF MS ES-

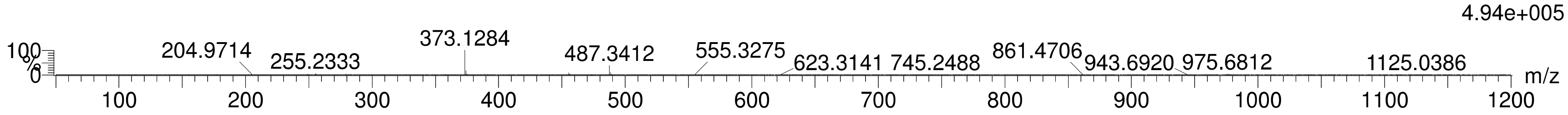

Minimum:				-1.5
Maximum:		20.0	10.0	50.0

Mass	Calc. Mass	mDa	PPM	DBE	i-FIT	Norm	Conf(%)	Formula
373.1284	373.1287	-0.3	-0.8	10.5	1444.6	n/a	n/a	C20 H21 O7

Figure S27. HRMS spectrum for compound **4**

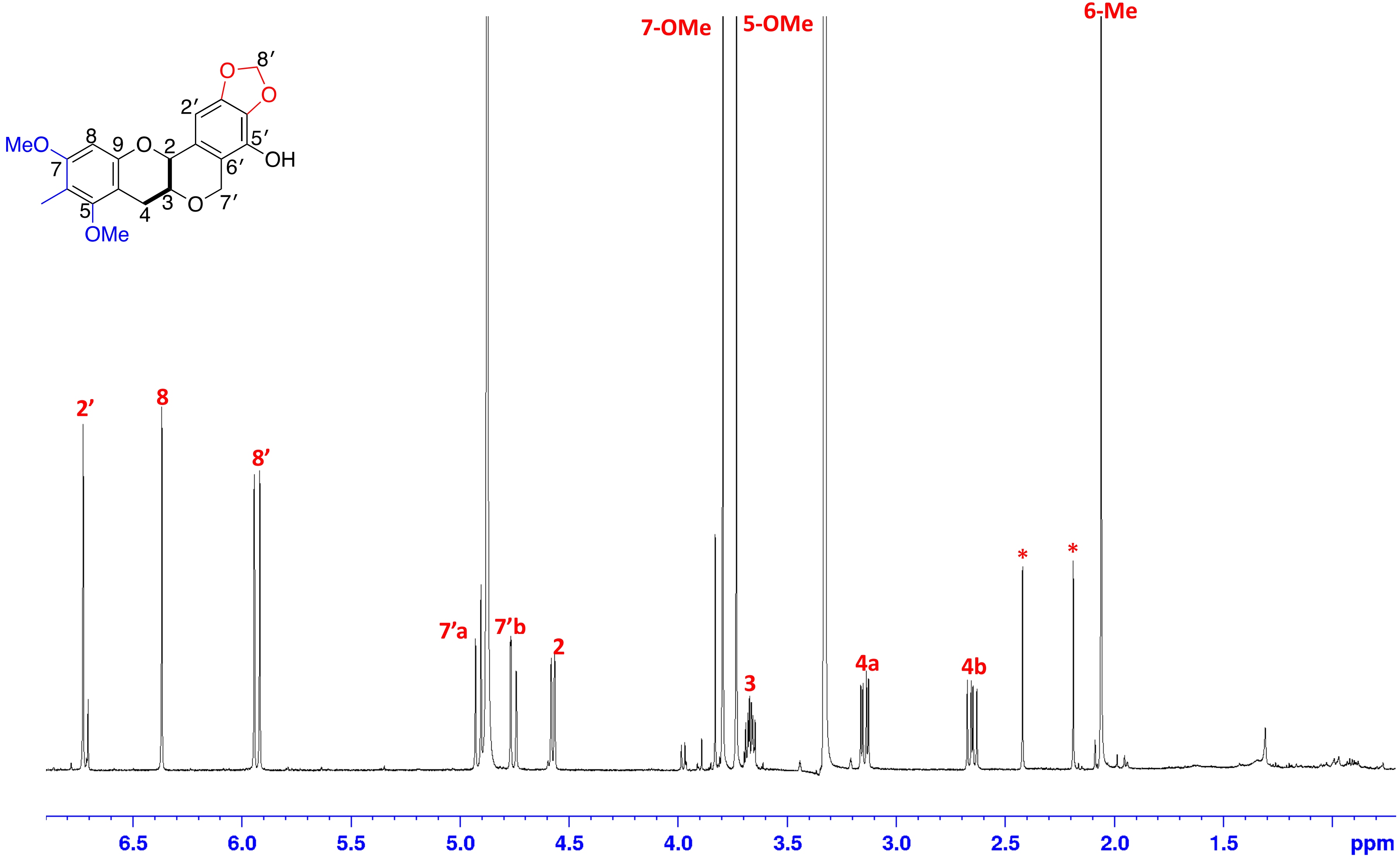

Figure S28. ^{1}H NMR spectrum of compound **5** (CD_3OD, 600 MHz)

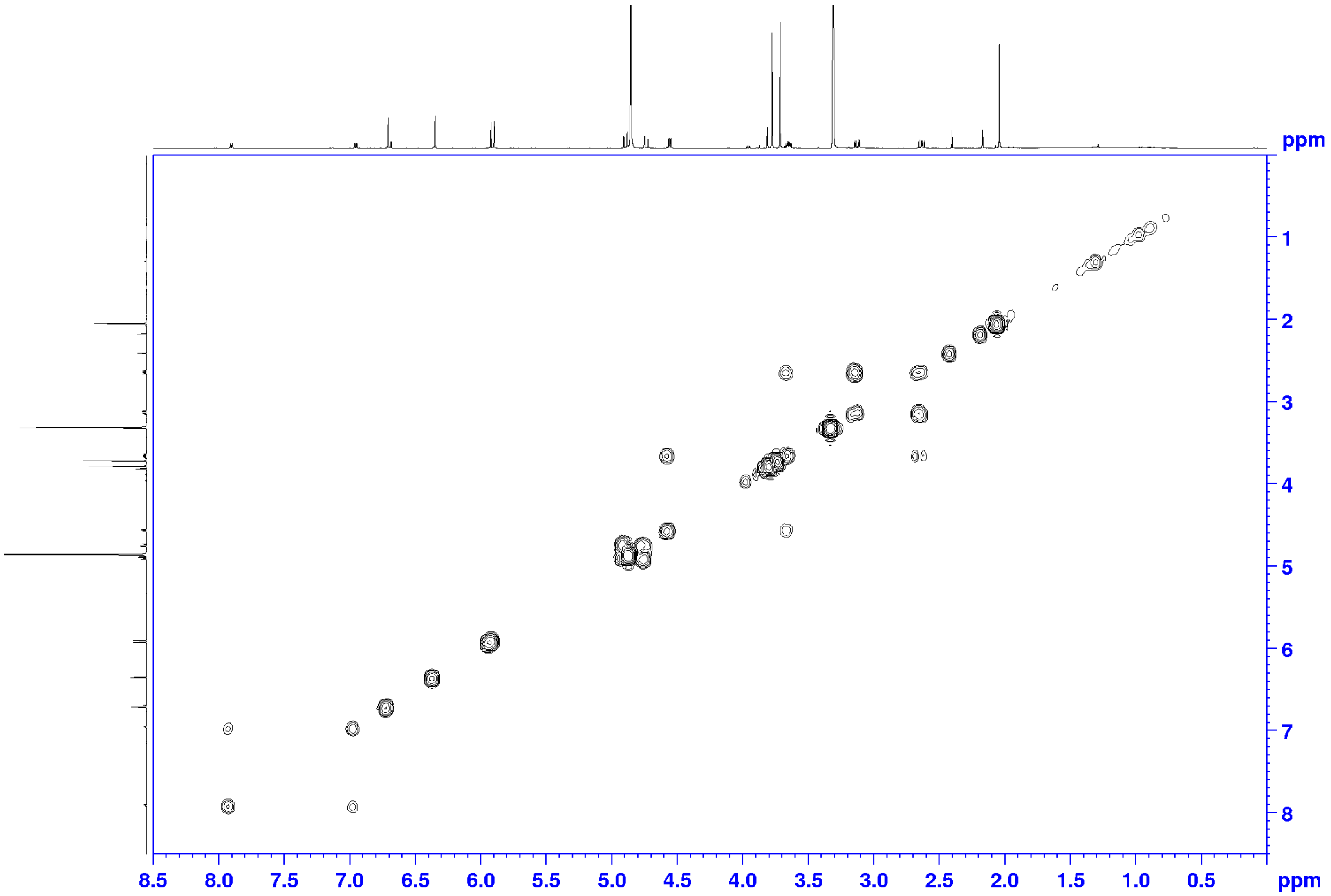

ppm
1
2
3
4
5
6
7
8
8.5
8.0
7.5
7.0
6.5
6.0
5.5
5.0
4.5
4.0
3.5
3.0
2.5
2.0
1.5
1.0
0.5
ppm

Figure S29. COSY spectrum of compound **5** (CD_3OD, 600 MHz)

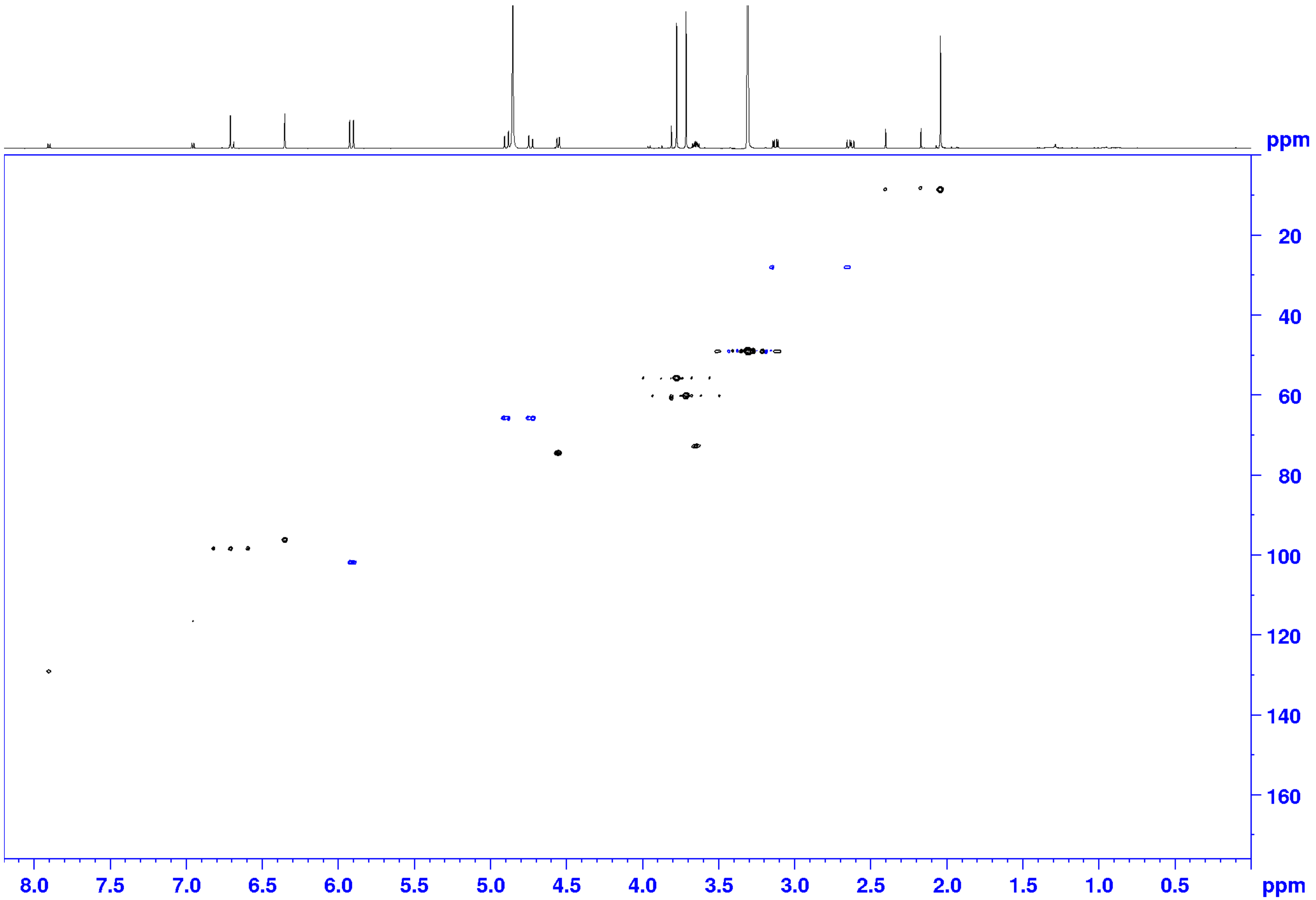

Figure S30. HSQC spectrum of compound **5** (CD_3OD, 600 MHz)

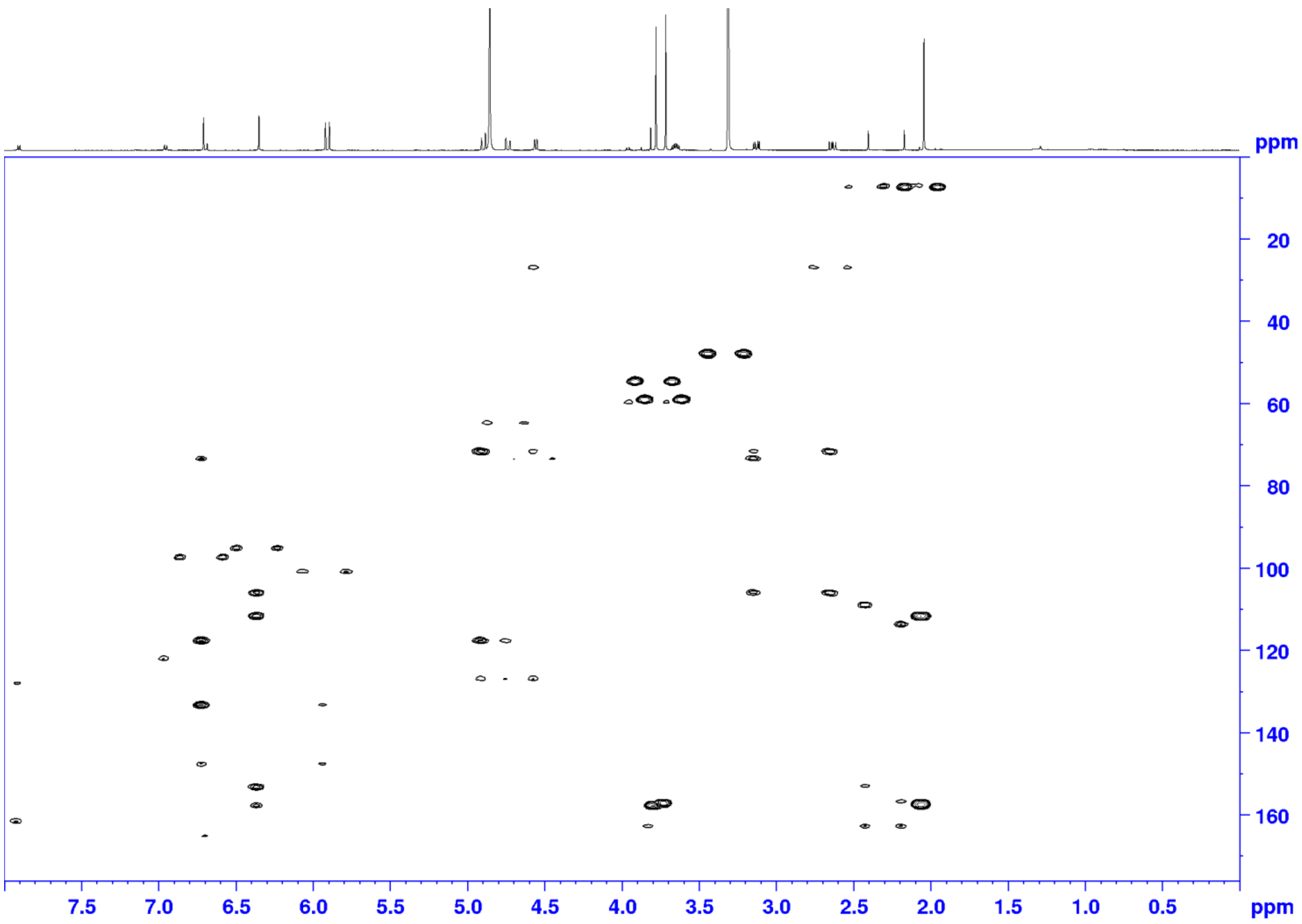

Figure S31. HMBC spectrum of compound **5** (CD_3OD, 600 MHz)

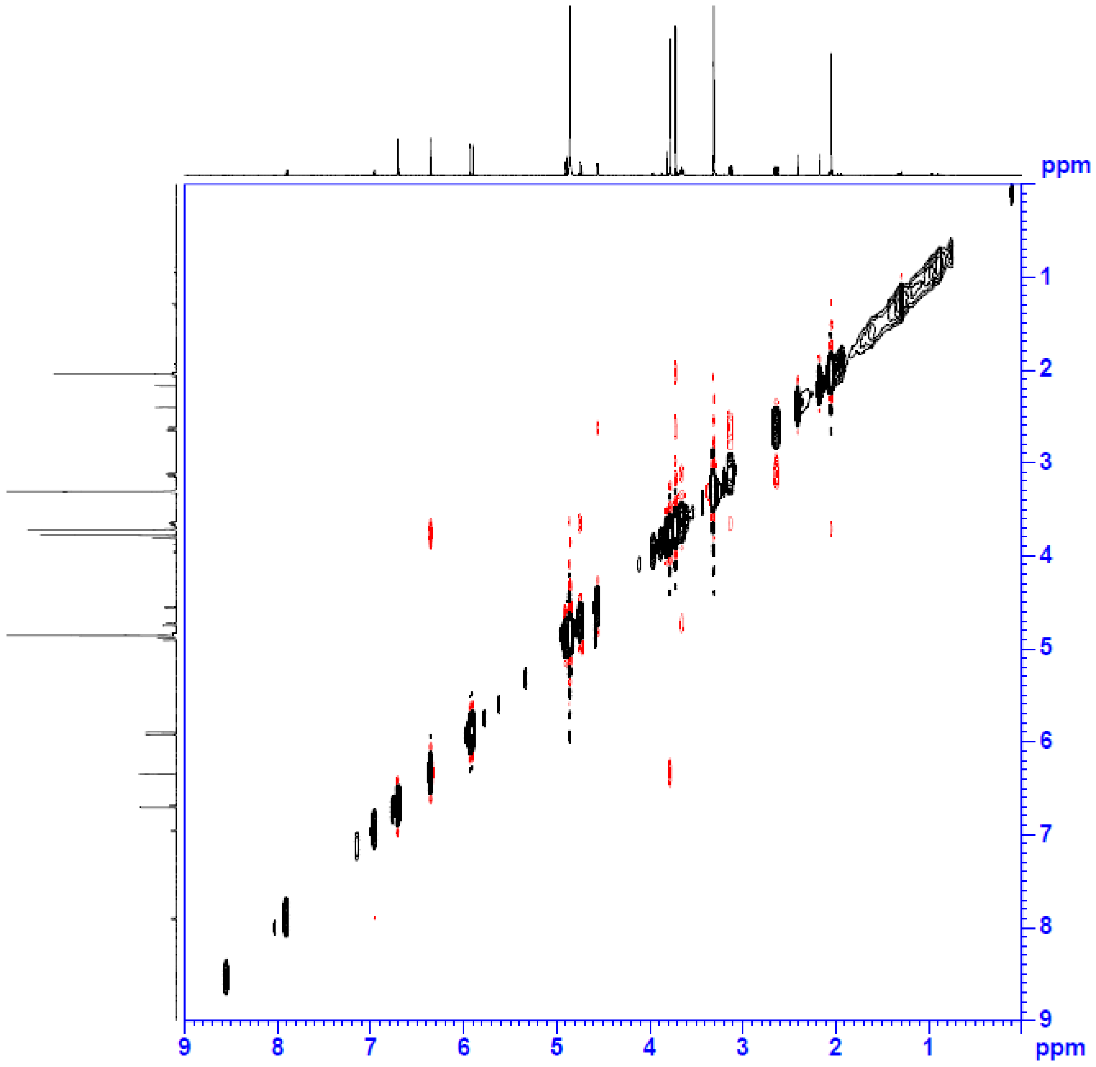

ppm
1
2
3
4
5
6
7
8
9
9
8
7
6
5
4
3
2
1
ppm

Figure S32. NOESY spectrum of compound **5** (CD_3OD, 600 MHz)

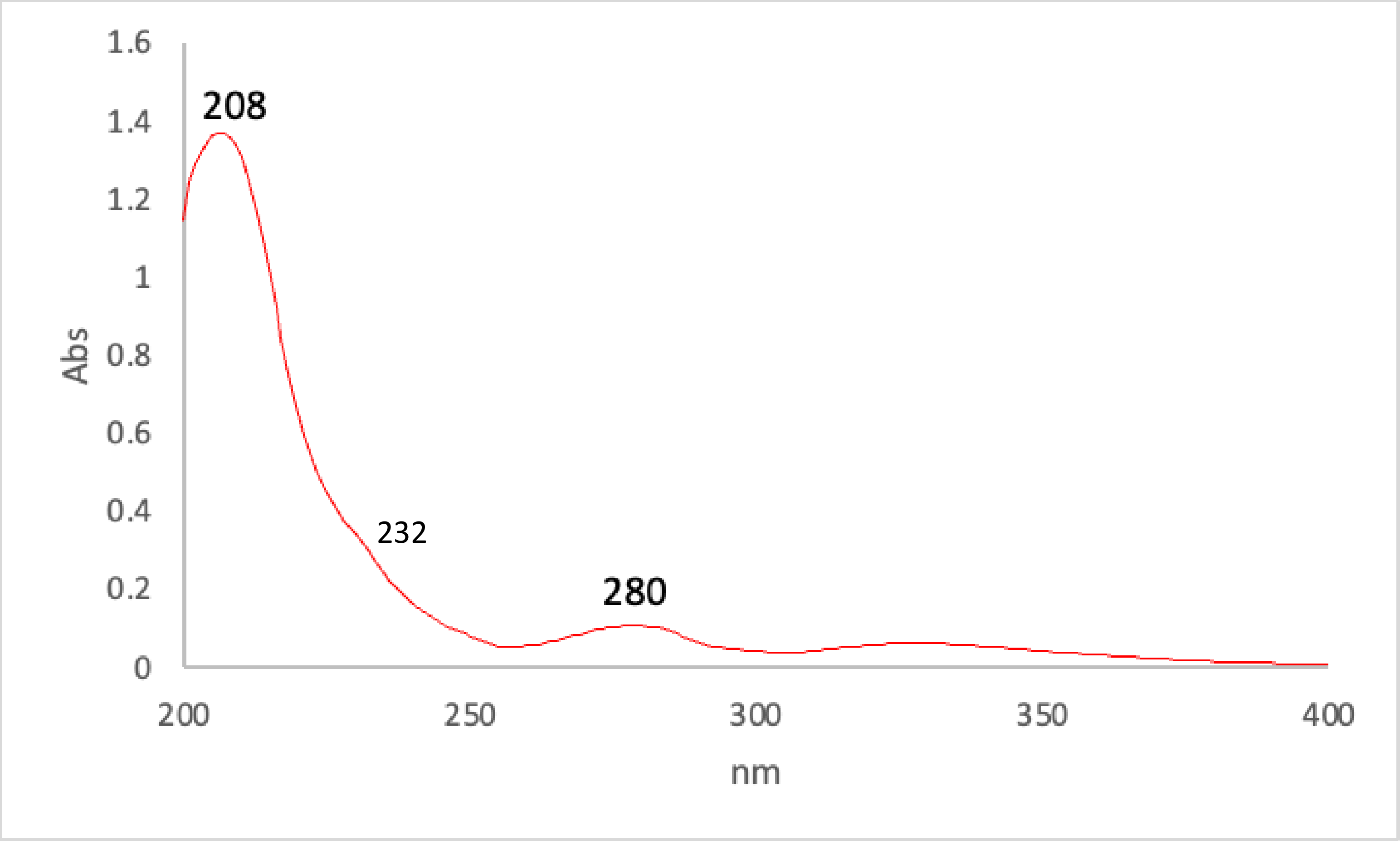

Figure S33. UV-Vis spectrum for compound **5**

Elemental Composition Report

Single Mass Analysis

Tolerance = 5.0 PPM / DBE: min = -1.5, max = 50.0
Element prediction: Off
Number of isotope peaks used for i-FIT = 9

Monoisotopic Mass, Even Electron Ions
15 formula(e) evaluated with 1 results within limits (all results (up to 1000) for each mass)
Elements Used:
C: 0-25 H: 0-30 O: 0-8
01122022_BMF-16B 122 (2.396) Cm (122)
1: TOF MS ES-

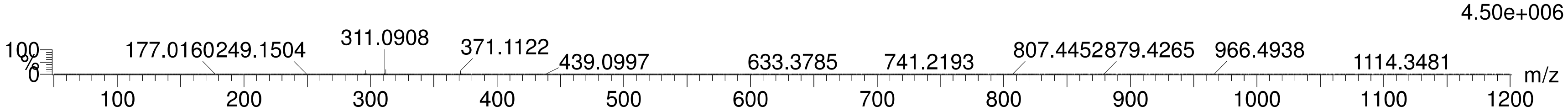

	Mass	Calc. Mass	mDa	PPM	DBE	i-FIT	Norm	Conf(%)	Formula
Minimum:					-1.5				
Maximum:			20.0	5.0	50.0				
	371.1122	371.1131	-0.9	-2.4	11.5	1273.8	n/a	n/a	C20 H19 O7

Figure S34. HRMS spectrum for compound **5**

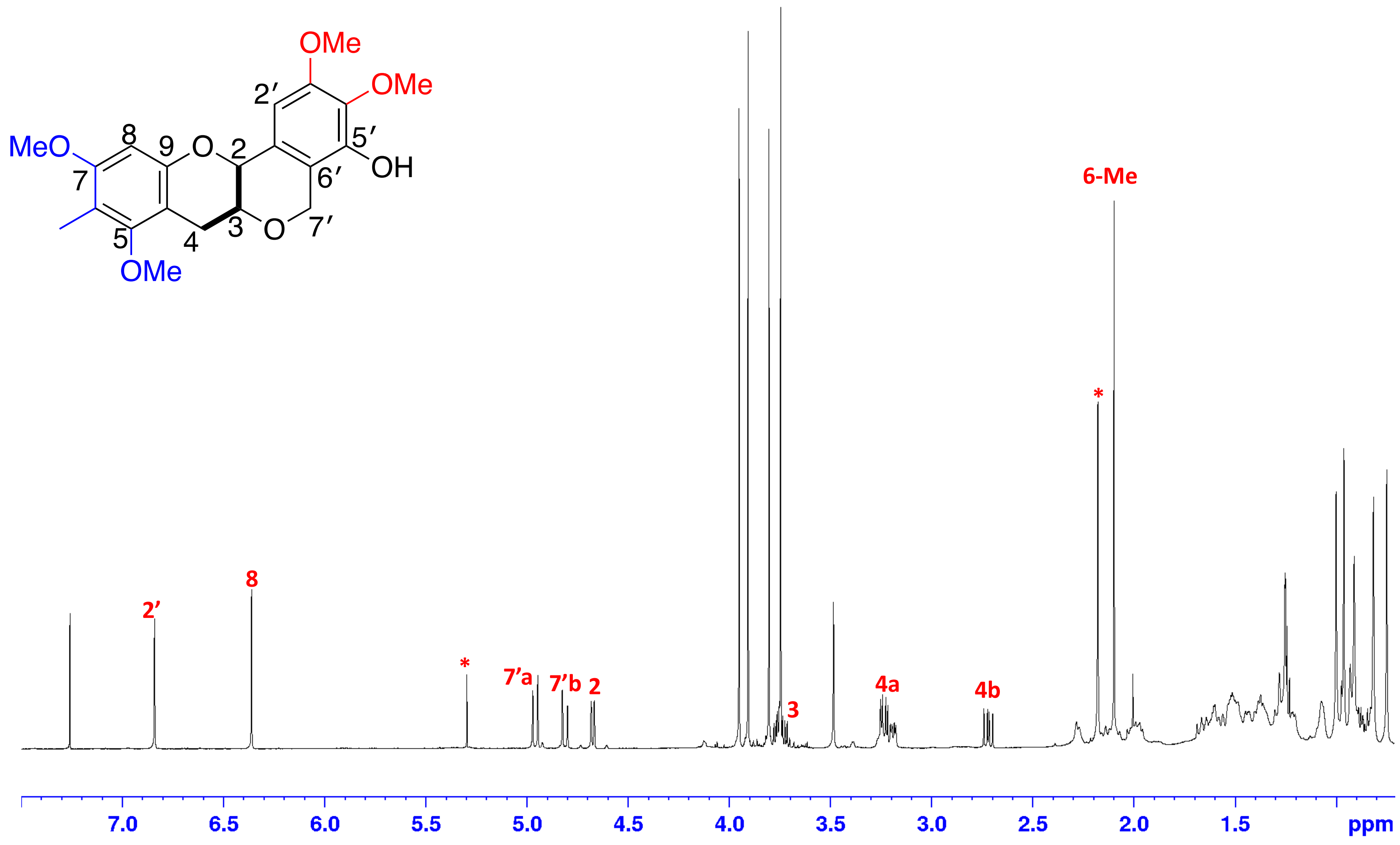

Figure S35. ^{1}H NMR spectrum of compound **6** ($CDCl_3$, 600 MHz)

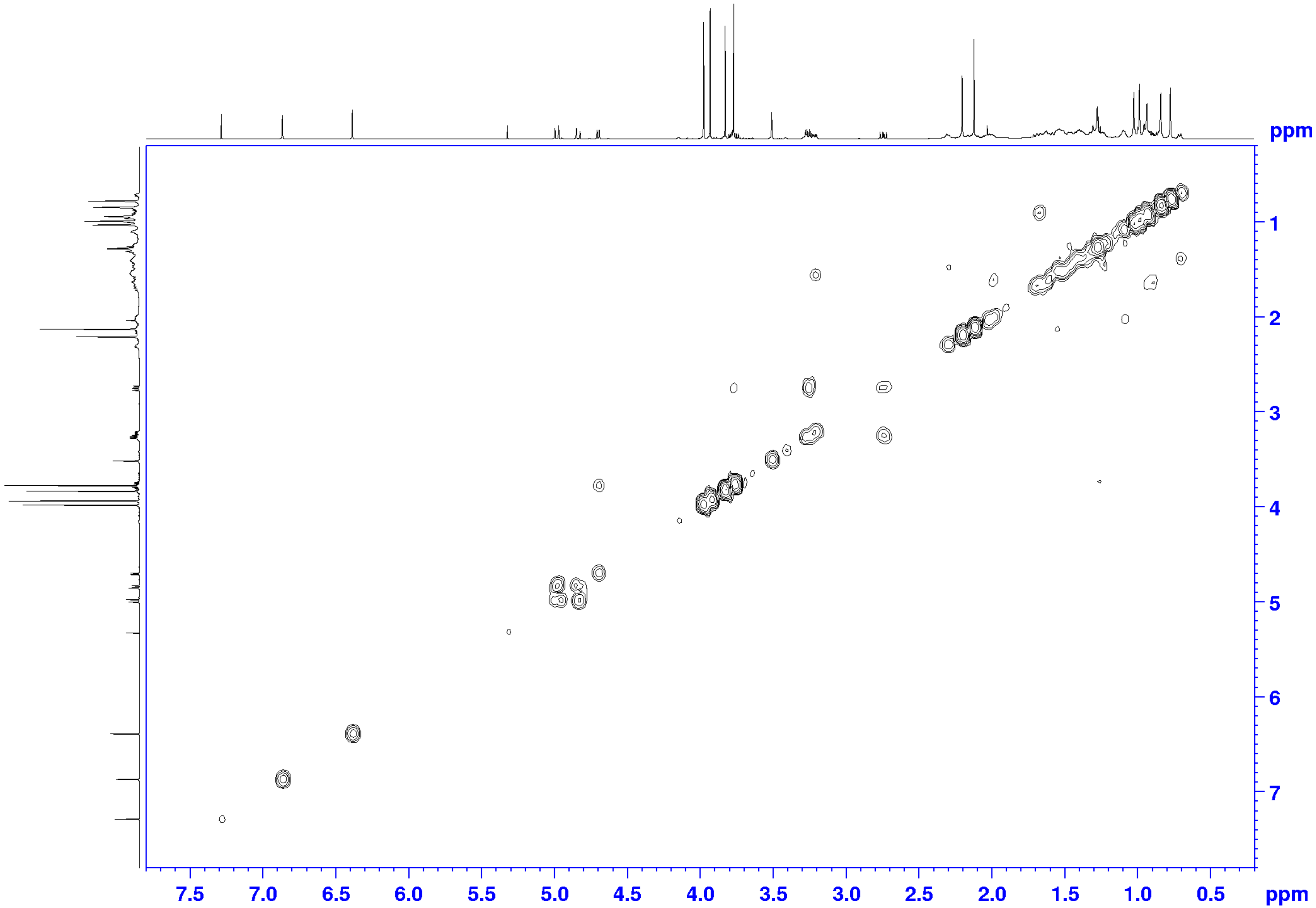

Figure S36. COSY spectrum of compound **6** ($CDCl_3$, 600 MHz)

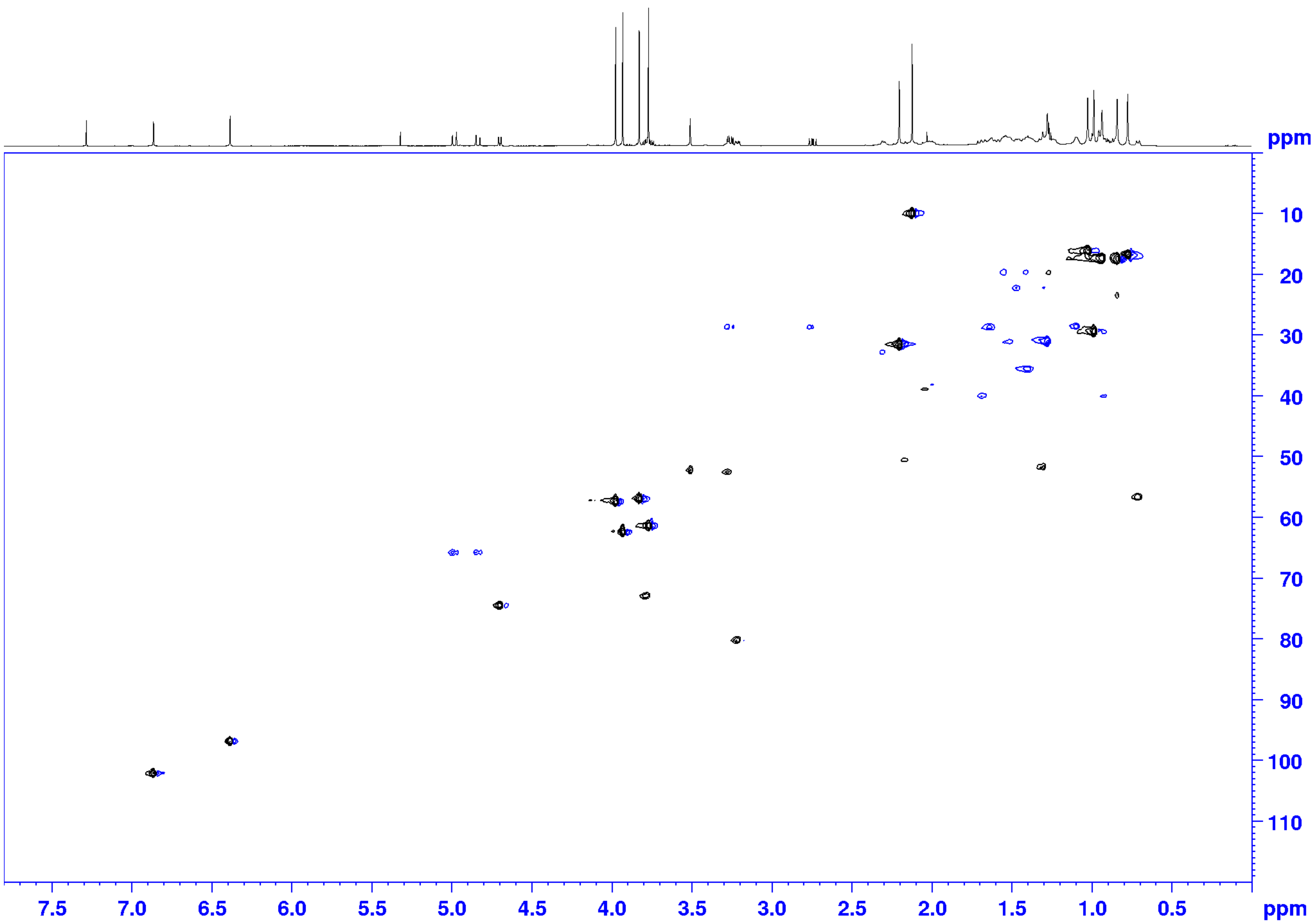

Figure S37. HSQC spectrum of compound **6** ($CDCl_3$, 600 MHz)

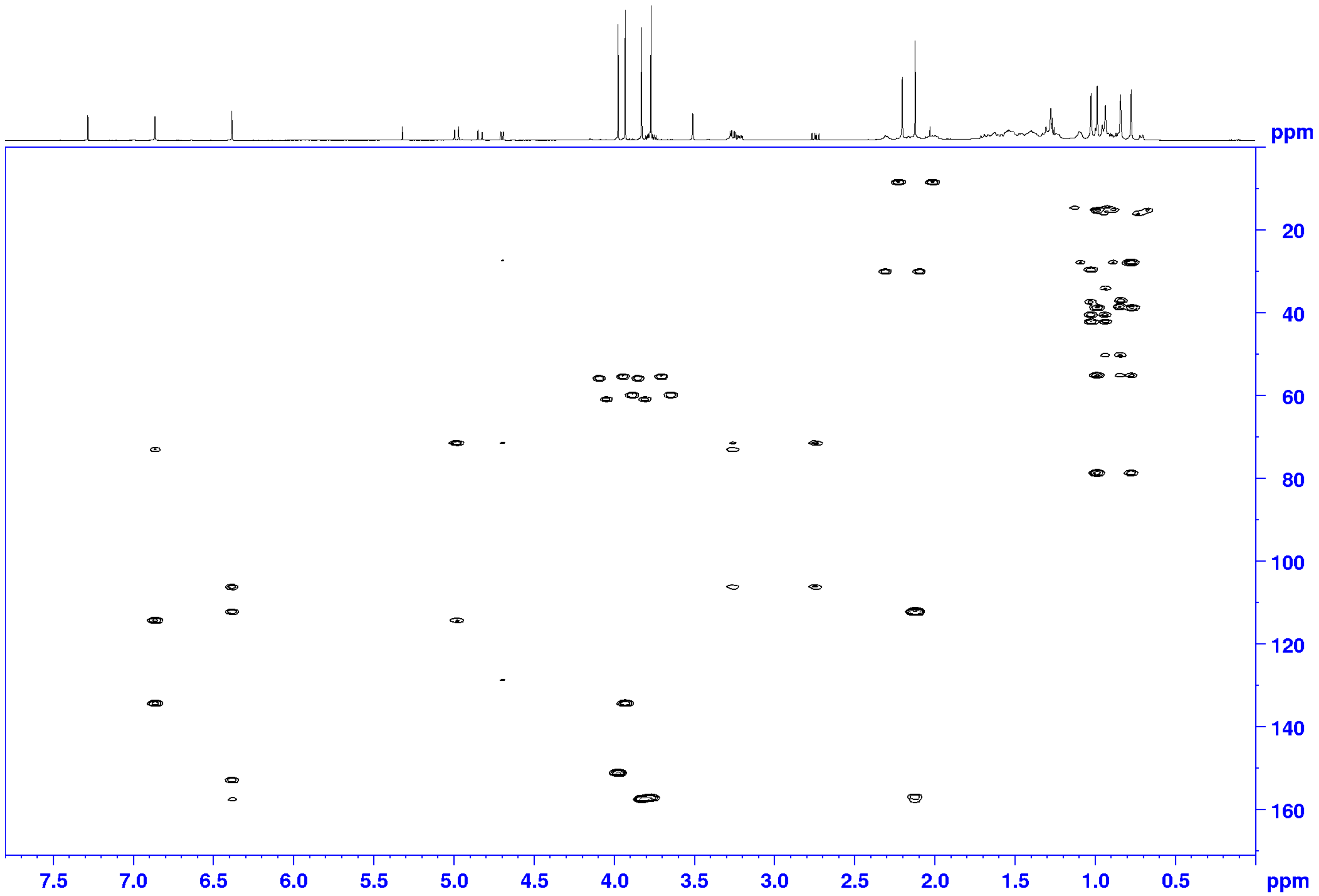

Figure S38. HMBC spectrum of compound **6** ($CDCl_3$, 600 MHz)

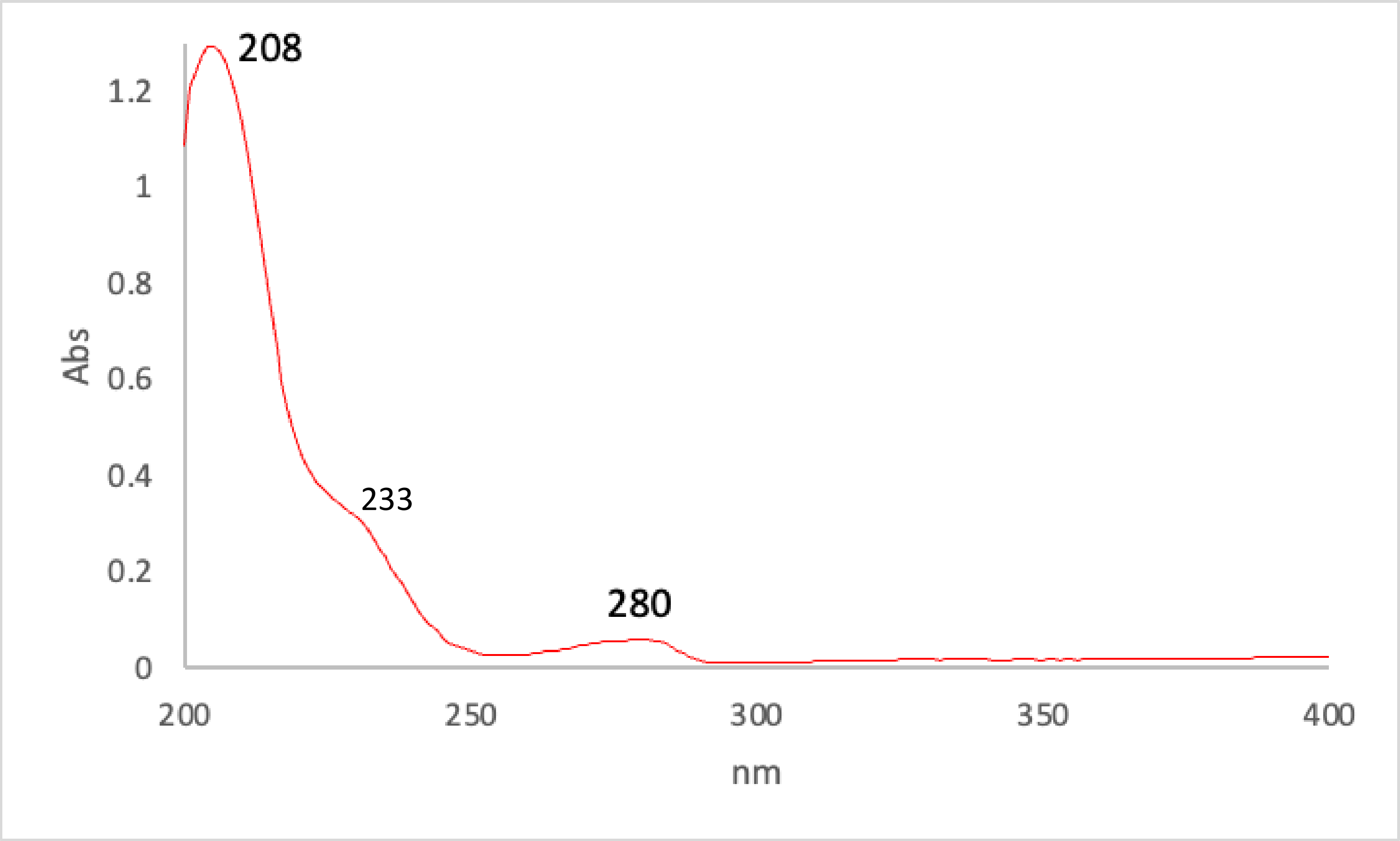

Figure S39. UV-Vis spectrum for compound **6**

Elemental Composition Report

Page 1

Single Mass Analysis

Tolerance = 10.0 PPM / DBE: min = -1.5, max = 50.0
Element prediction: Off
Number of isotope peaks used for i-FIT = 9

Monoisotopic Mass, Even Electron Ions
7 formula(e) evaluated with 1 results within limits (all results (up to 1000) for each mass)
Elements Used:
C: 0-21 H: 0-24 O: 0-7

221103_BM_F17A_ 17 (0.364) Cm (17:18)
1: TOF MS ES-

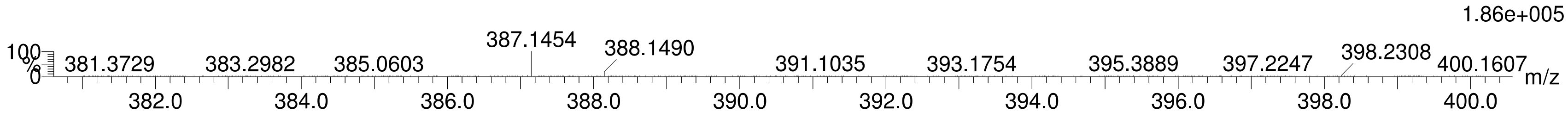

Minimum:				-1.5				
Maximum:		20.0	10.0	50.0				
Mass	Calc. Mass	mDa	PPM	DBE	i-FIT	Norm	Conf(%)	Formula
387.1454	387.1444	1.0	2.6	10.5	1577.3	n/a	n/a	C21 H23 O7

Figure S40. HRMS spectrum for compound **6**

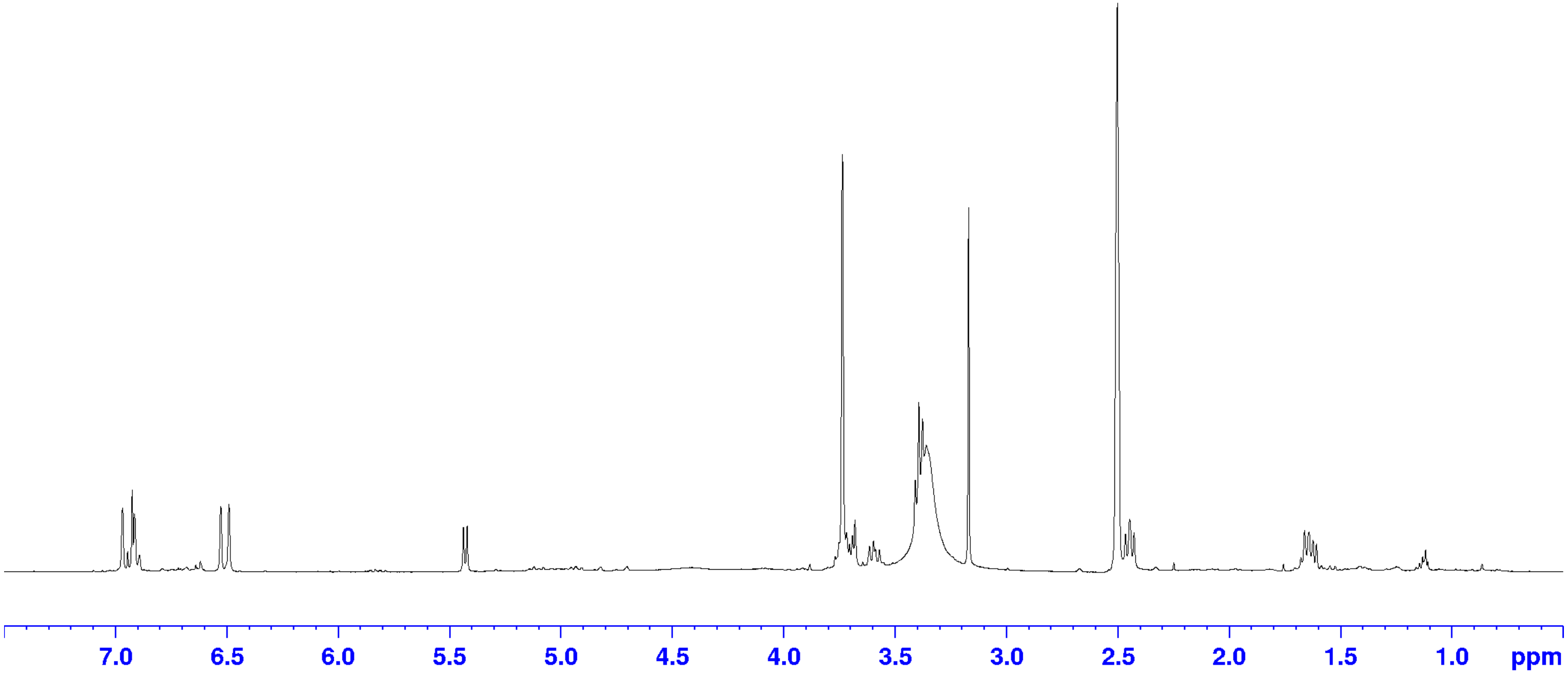

Figure S41. ^{1}H NMR spectrum of compound **7** (DMSO-d_6, 400 MHz)

Elemental Composition Report

Single Mass Analysis

Tolerance = 5.0 PPM / DBE: min = -1.5, max = 50.0
Element prediction: Off
Number of isotope peaks used for i-FIT = 9

Monoisotopic Mass, Even Electron Ions
16 formula(e) evaluated with 1 results within limits (all results (up to 1000) for each mass)
Elements Used:
C: 0-25 H: 0-30 O: 0-8
011222_BMF-4 122 (2.396) Cm (122)
1: TOF MS ES-

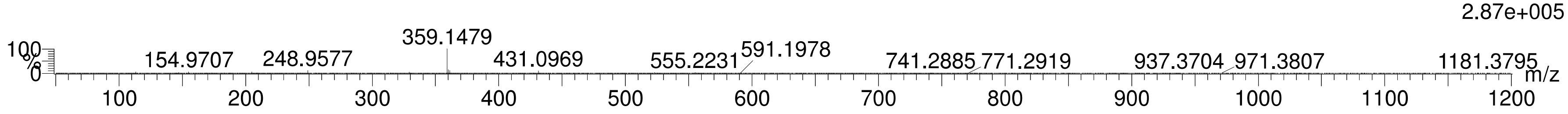

	Mass	Calc. Mass	mDa	PPM	DBE	i-FIT	Norm	Conf(%)	Formula
Minimum:					-1.5				
Maximum:			20.0	5.0	50.0				
	359.1479	359.1495	-1.6	-4.5	9.5	1187.0	n/a	n/a	C20 H23 O6

Figure S42. HRMS spectrum for compound **7**

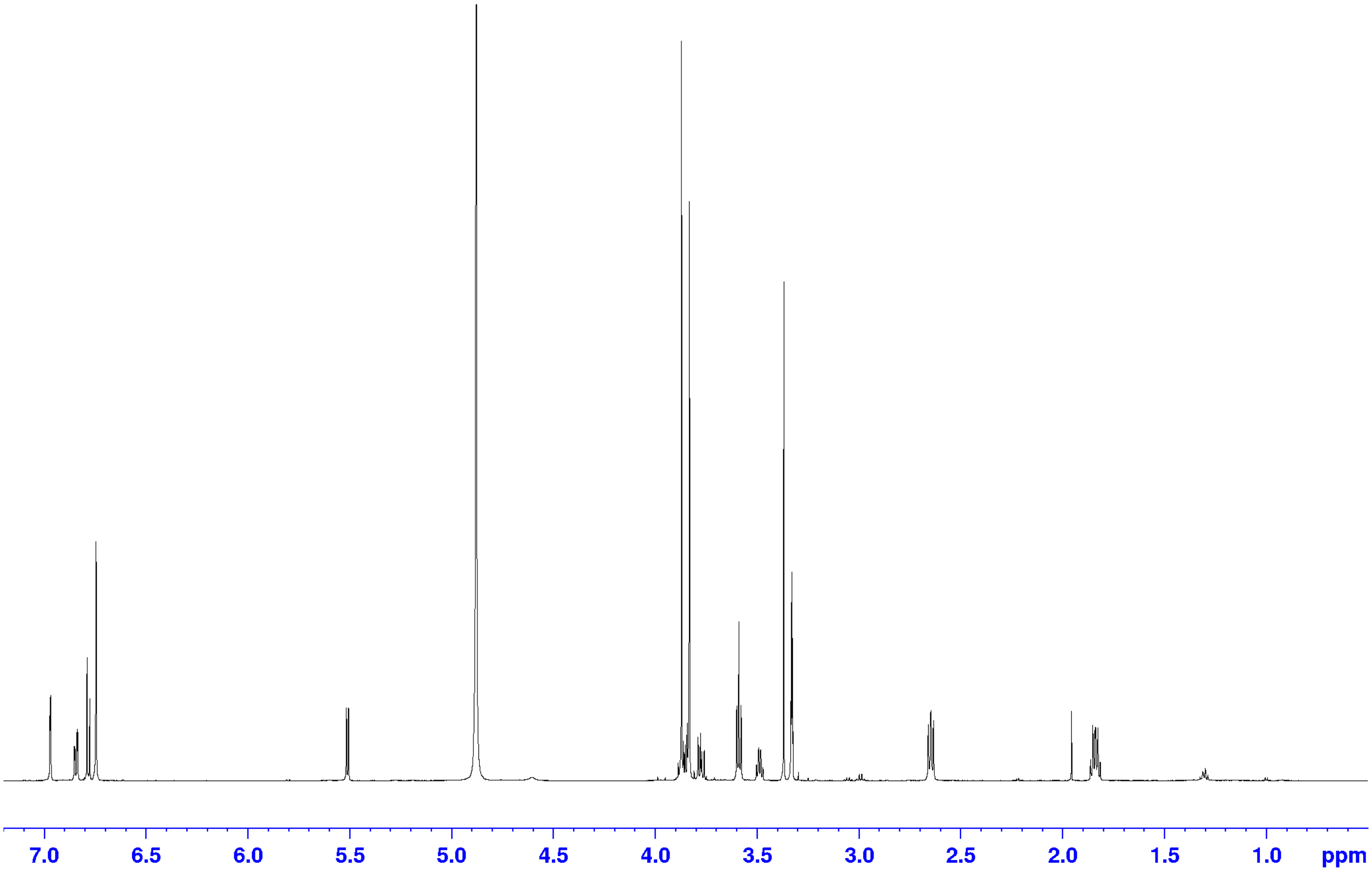

Figure S43. ^{1}H NMR spectrum of compound **8** (CD_3OD, 600 MHz)

Elemental Composition Report

Single Mass Analysis

Tolerance = 20.0 PPM / DBE: min = -1.5, max = 50.0
Element prediction: Off
Number of isotope peaks used for i-FIT = 9

Monoisotopic Mass, Even Electron Ions
70 formula(e) evaluated with 2 results within limits (all results (up to 1000) for each mass)
Elements Used:
C: 0-50 H: 0-50 O: 0-20

251222BMF-4A_1 negattive LS 73 (1.449)
1: TOF MS ES-

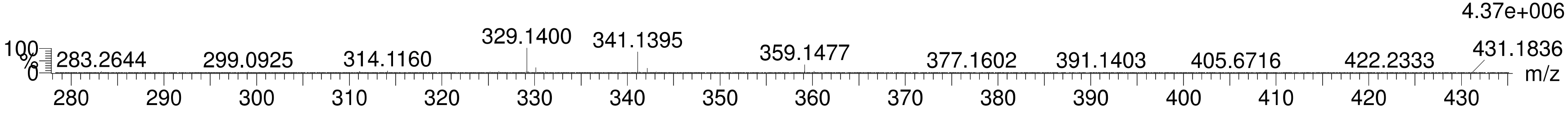

Minimum:				-1.5				
Maximum:		20.0	20.0	50.0				
Mass	Calc. Mass	mDa	PPM	DBE	i-FIT	Norm	Conf(%)	Formula
359.1477	359.1495	-1.8	-5.0	9.5	2548.8	0.000	99.99	C20 H23 O6
	359.1436	4.1	11.4	18.5	2558.6	9.820	0.01	C27 H19 O

Figure S44. HRMS spectrum for compound **8**

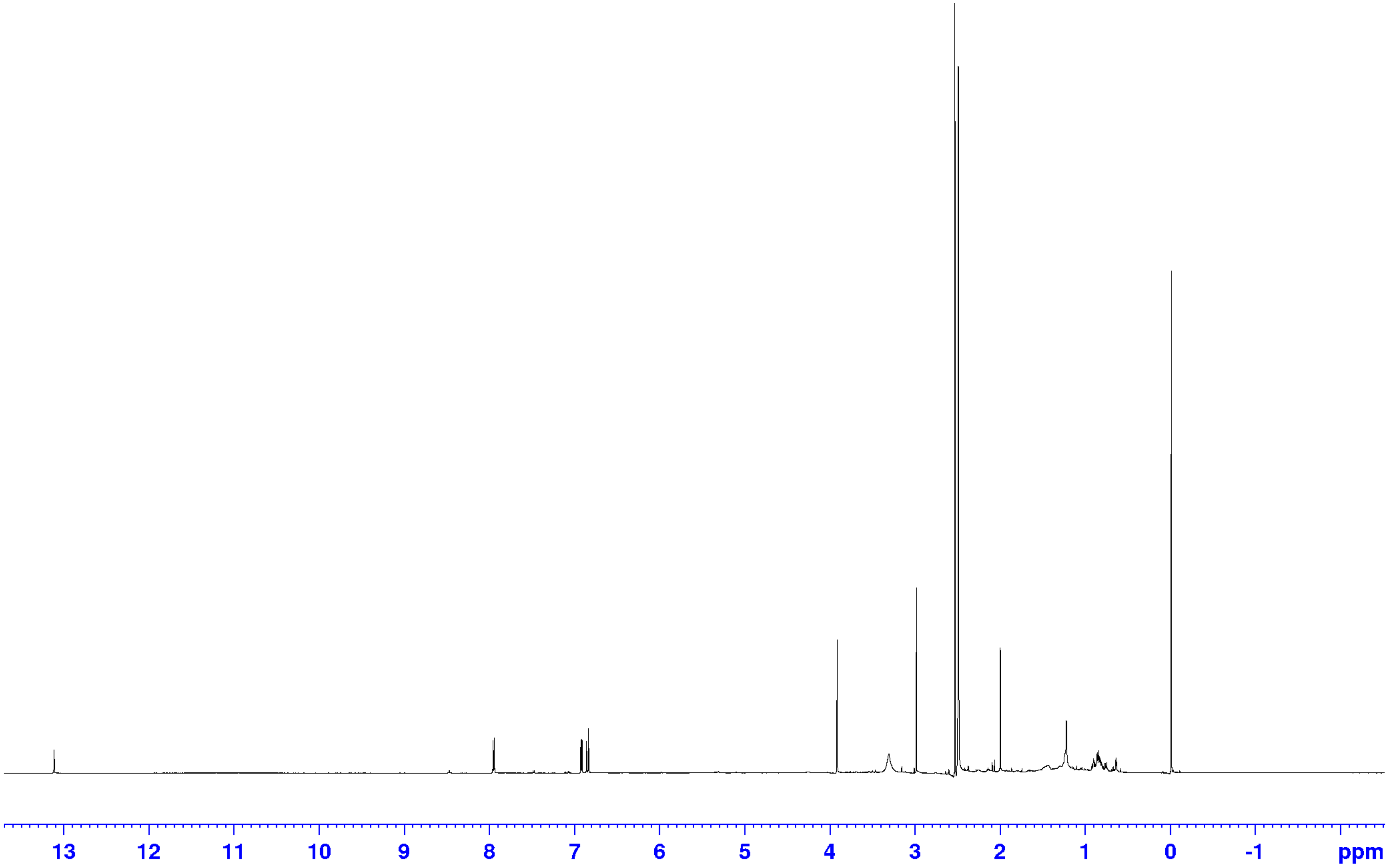

Figure S45. ^{1}H NMR spectrum of compound **9** (DMSO-d$_6$, 600 MHz)

Elemental Composition Report

Single Mass Analysis

Tolerance = 5.0 PPM / DBE: min = -1.5, max = 50.0
Element prediction: Off
Number of isotope peaks used for i-FIT = 9

Monoisotopic Mass, Even Electron Ions
24 formula(e) evaluated with 1 results within limits (all results (up to 1000) for each mass)
Elements Used:
C: 0-25 H: 0-30 O: 0-8
011222_BMF-15 122 (2.396) Cm (122)
1: TOF MS ES-

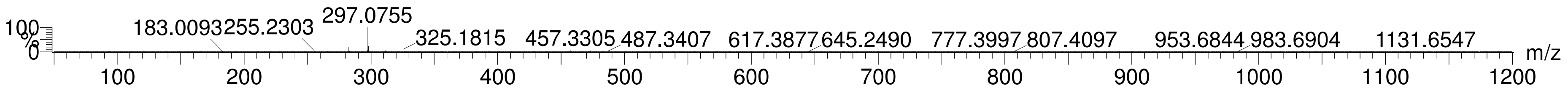

Minimum:				-1.5				
Maximum:		20.0	5.0	50.0				
Mass	Calc. Mass	mDa	PPM	DBE	i-FIT	Norm	Conf(%)	Formula
297.0755	297.0763	-0.8	-2.7	11.5	2162.9	n/a	n/a	C17 H13 O5

Figure S46. HRMS spectrum for compound **9**

Table S2. NMR data (600 MHz, CD_3OD) for compound **1**

Position	$\delta_{H,}$ mult, (*J* in Hz)	*δ_C (ppm)	COSY	HMBC
2	4.53 d (*9.0*)	74.6	3	3, 4, 1′, 6′
3	3.64 m	73.1	2,4	
4	3.11 dd (*15.5, 6.0*)<br>2.63 dd (*15.5, 10.7*)	28.2	3	3, 5, 9,1 0
5		157.6		
6		110.5		
7		155.2		
8	6.21 s	99.7		6, 7, 9, 10
9		152.9		
10		105.1		
5-OMe	3.71 s	60.3		5
6-Me	2.05 s	8.6		5, 6, 7
7-OMe				
1′		117.7		
2′	6.66 s	98.4		1′, 2, 3′, 4′
3′		133.4		
4′		147.6		
5′		135.7		
6′		126.9		
7′	4.89 d (*15.2*)<br>4.73 d (*15.2*)	65.8		3, 1′, 5′, 6′
8′	5.91 d (*15.3*)	101.9		3′, 4′
3′ OMe				
4′ OMe				

*assignments supported by HSQC and HMBC experiments

Table S3. NMR data (600 MHz, CD_3OD) for compound **2**

Position	δ_H, mult, (*J* in Hz)	*δ_C (ppm)	COSY	HMBC	NOESY
2	4.57 d (*9.5*)	74.4	3	3, 4, 1′, 2′, 6′	
3	3.67 ddd (*10.7, 9.5, 6.1*)	72.9	2, 4		7′
4	3.15 dd (*15.4, 6.1*)<br>2.66 dd (*15.4, 10.7*)	28.1	3	2, 3, 5, 9, 10	
5		158.8			
6		111.6			
7		156.2			
8	6.23 s	99.4		6,7,9,10	
9		154.0			
10		106.8			
5-OMe	3.72 s	60.2		5	6-Me
6-Me	2.07 s	8.4		5,6,7	5-OMe
7-OMe					
1′		116.0			
2′	6.78 s	101.3		2, 1′, 3′, 4′	3′ OMe
3′		136.2			
4′		153.2			
5′		146.4			
6′		129.7			
7′	4.88 (*15.3*)<br>4.73 (*15.3*)	65.5		3, 1′, 5′, 6′	3
8′		101.3			
3′ OMe	3.88 s	56.3		3′	2′
4′ OMe	3.79 s	60.9		4′	

*assignments supported by HSQC and HMBC experiments

Table S4. NMR data (600 MHz, CD_3OD) for compound **3**

Position	δ_H, mult, (*J* in Hz)	*δ_C (ppm)	COSY	HMBC	NOESY
2	4.74 d (*9.9*)	72.7	3	3, 1′, 6′	
3	3.67 dd (*9.9, 8.3*)	78.3	2,4		
4	5.01 d (*8.3*)	70.6	3	3,9,10	
5		156.8			
6		106.8			
7		160.0			
8	6.15 s	92.1		6,7,9,10	7-OMe
9		154.0			
10		103.8			
5-OMe					
6-Me	1.98 s	7.7		5,6,7	
7-OMe	3.77 s	55.8		7	8
1′		116.1			
2′	6.79 s	101.4		2, 1′, 3′, 4′, 6′	3′ OMe
3′		135.5			
4′		153.2			
5′		147.0			
6′		129.4			
7′	4.92 d (*15.1*)<br>4.72 d (*15.1*)	65.6		3, 1′, 5′, 6′	
8′					
3′ OMe	3.89 s	56.2		3′	2′
4′ OMe	3.81 s	60.8		4′	

*assignments supported by HSQC and HMBC experiments

Table S5. NMR data (600 MHz, CD_3OD) for compound **4**

Position	δ_H, mult, (*J* in Hz)	*δ_C (ppm)	COSY	HMBC	NOESY
2	4.56 d (*9.6*)	74.2	3	3, 1′, 6′	
3	3.71 ddd (*10.5, 9.6, 6.1*)	73.3	2,4		7′
4	3.10 dd (*15.4, 6.1*)<br>2.57 dd (*15.4, 10.5*)	28.6	3	3,9,10	
5		158.8			
6		106.5			
7		158.5			
8	6.19 s	92.4		6,7,9,10	
9		154.2			
10		102.8			
5-OMe					
6-Me	2.02 s	8.1		5,6,7	7-OMe
7-OMe	3.76 s	55.8		7	6-Me
1′		116.3			
2′	6.83 s	101.0		2, 1′, 3′, 4′, 6′	3′ OMe
3′		136.2			
4′		153.3			
5′		147.1			
6′		130.1			
7′	4.89 d (*14.0*)<br>4.74 d (*15.1*)	65.5		3, 1′, 5′, 6′	3
8′					
3′ OMe	3.89 s	56.2		3′	2′
4′ OMe	3.79 s	60.9		4′	

*assignments supported by HSQC and HMBC experiments

Table S6. NMR data (600 MHz, CD_3OD) for compound **5**

Position	$\delta_{H,}$ mult, (*J* in Hz)	*δ_C (ppm)	COSY	HMBC	NOESY
2	4.58 d (*9.1*)	74.6	3	3, 1′, 6′	
3	3.67 ddd (*10.6, 9.1, 5.9*)	72.7	2,4		
4	3.14 dd (*15.4, 5.9*)<br>2.65 dd (*15.4, 10.6*)	28.0	3	2,3,5, 9,10	
5		158.3			
6		112.8			
7		158.8			
8	6.36 s	101.9		6,7,9,10	7-OMe
9		154.8			
10		108.0			
5-OMe	3.73 s	60.1			6-Me
6-Me	2.06 s	8.7		5,6,7	5-OMe
7-OMe	3.80 s	55.7		7	8
1′		118.4			
2′	6.72 s	98.6		2, 1′, 3′, 4′, 6′	
3′		134.9			
4′		148.8			
5′		137.5			
6′		128.4			
7′	4.91 d (*15.2*)<br>4.76 d (*15.2*)	65.8		3, 1′, 5′, 6′	
8′	5.93 d (*14.0*)	101.9		3′, 4′	
3′ OMe					
4′ OMe					

*assignments supported by HSQC and HMBC experiments

Table S7. NMR data (600 MHz, $CDCl_3$) for compound **6**

Position	$\delta_{H,}$ mult, (*J* in Hz)	*δ_C (ppm)	COSY	HMBC
2	4.67 d (*9.5*)	74.3	3	3, 1′, 6′
3	3.74 m	72.8	2,4	
4	3.23 dd (*15.3*, *6.0*)<br>2.71 dd (*15.3*, *10.6*)	28.4	3	2,3,5, 9,10
5		157.4		
6		112.6		
7		157.8		
8	6.36 s	99.6		6,7,9,10
9		153.1		
10		106.8		
5-OMe	3.73 s	61.1		5
6-Me	2.09 s	9.4		5,6,7
7-OMe	3.80 s	56.7		7
1′		114.2		
2′	6.84 s	102.4		2, 1′, 3′, 4′
3′		134.4		
4′		147.7		
5′				
6′		128.7		
7′	4.96 d (*15.4*)<br>4.81 d (15.4)	65.7		3, 1′, 4′, 5′, 6′
8′				
3′ OMe	3.95 s	57.1		3′
4′ OMe	3.91 s	62.2		4′

*assignments supported by HSQC and HMBC experiments

Table S8. Downregulation of LPS and IFN- γ induced nitric oxide production and cell viability of sequential extracts

Sequential Extracts	Inhibition of NO production (μg/mL), N=6	Cell viability (μg/mL), N=6
Hexane	39.11±6.82	>100
DCM	14.09±0.81	30.65±2.43
EtOAc	18.25±7.60	>100
Ethanol	69.25±13.33	>100
MeOH	49.93 ± 8.76	>100
Water	80.25±17.18	>100
